# TMEM44-AS1 promotes esophageal squamous cell carcinoma progression by regulating the IGF2BP2-GPX4 axis in modulating ferroptosis

**DOI:** 10.1038/s41420-023-01727-0

**Published:** 2023-12-01

**Authors:** Ruotong Yang, Junhu Wan, Liwei Ma, Fuyou Zhou, Zhengwu Yang, Zhuofang Li, Mingyuan Zhang, Liang Ming

**Affiliations:** 1 Department of Clinical Laboratory, The First Affiliated Hospital of Zhengzhou University, Zhengzhou, China; 2 Key Clinical Laboratory of Henan province, Zhengzhou, China; 3 Thoracic Department, Anyang Tumor Hospital, Henan Key Medical Laboratory of Precise Prevention and Treatment of Esophageal Cancer, Anyang, China

**Keywords:** Cell death, Long non-coding RNAs

## Abstract

The long non-coding RNA (lncRNA) TMEM44-AS1 is a novel lncRNA whose pro-carcinogenic role in gastric cancer and glioma has been demonstrated. However, its function in esophageal squamous cell carcinoma (ESCC) is unknown. In this study, we identified that TMEM44-AS1 was highly expressed in ESCC tissues and cells. Functionally, TMEM44-AS1 promoted ESCC cell proliferation, invasion and metastasis in vitro and in vivo. TMEM44-AS1 inhibited ferroptosis in ESCC cells, and ferroptosis levels were significantly increased after knockdown of TMEM44-AS1. Mechanistically, TMEM44-AS1 was positively correlated with GPX4 expression, and TMEM44-AS1 could bind to the RNA-binding protein IGF2BP2 to enhance the stability of GPX4 mRNA, thereby affecting ferroptosis and regulating the malignant progression of ESCC. In summary, this study reveals the TMEM44-AS1-IGF2BP2-GPX4 axis could influence cancer progression in ESCC. TMEM44-AS1 can be used as a potential treatment target against ESCC.

## Introduction

Esophageal cancer (EC) is the seventh most common malignancy and the sixth leading cause of cancer related death in the world [1]. Despite many advances in diagnosis and treatment in recent years, patients diagnosed with EC have an extremely poor 5-year survival rate of only 15% - 20% [2, 3]. According to histological subtypes, EC is divided into esophageal squamous cell carcinoma and esophageal adenocarcinoma. Among the 456000 cases of EC that occur every year, ESCC accounts for about 90% [4]. Current treatment options for EC include multimodal therapy, which includes surgery, radiotherapy and chemotherapy. Tumor markers of EC is a progressive research field, which may lead to early diagnosis [5]. Identifying susceptibility genes and biomarkers can help predict the treatment response of patients and improve the survival rate of patients [6].

LncRNAs are defined as non-coding RNAs longer than 200 nucleotides in length. They are transcribed by RNA polymerase II, which are usually located in the nucleus and play roles in the cytoplasm. They carry out a variety of functions including nuclear functions such as regulating gene expression in cis or in trans, splicing regulation, and the nucleation of subnuclear domains [7, 8]. LncRNAs are emerging as important regulators in gene expression networks by controlling nuclear architecture and transcription in the nucleus and by modulating mRNA stability, translation and post-translational modifications in the cytoplasm [9]. Meanwhile, depending on their localization and their specific interactions with DNA, RNA and proteins, lncRNAs can modulate chromatin function, regulate the assembly and function of membraneless nuclear bodies, and interfere with signaling pathways [10]. For example, Chen et al. found that SNHG17 acts as an endogenous “sponge”, competing with miR338-3p to regulate SOX4, thus promoting the progression of EC [11]. Likewise, LncRNA CASC9 promotes esophageal squamous cell carcinoma metastasis by interacting with the CREB-binding protein and modifying histone acetylation to up-regulate LAMC2 expression [12]. The expression of lncRNAs in tumors is often dysregulated, so they may become potential therapeutic and prognostic targets.

The concept of ferroptosis was first pointed out by Dixon in 2012 and identified as a new way of cell death [13]. The main causes of ferroptosis are massive iron accumulation and lipid peroxidation, which is a type of cell death that is dependent on iron and reactive oxygen species. Iron inducible factor can directly or indirectly affect the content of glutathione peroxidase through different ways, resulting in the accumulation of ROS and the decline of antioxidant capacity, and finally lead to oxidative cell death [14]. This unique modality of cell death, driven by iron-dependent phospholipid peroxidation, is regulated by multiple cellular metabolic events, including redox homeostasis, iron handling, mitochondrial activity, and metabolism of amino acids, lipids, and sugars, in addition to numerous signaling pathways relevant to disease [15]. The morphological characteristics of ferroptosis mainly include the reduction or disappearance of mitochondrial cristae, the rupture of mitochondrial outer membrane and the concentration of mitochondrial membrane. These cell abnormalities are caused by strong membrane lipid peroxidation and loss of selective permeability of plasma membrane caused by oxidative stress [16, 17]. This kind of ferroptotic injury plays an important role in the fate of tumor cells via damage-related molecular patterns and multiple cancer pathways [18, 19]. For example, Lu et al. found that PVT1 regulated ferroptosis through miR-214-mediated TFR1 and TP53 expression. There was a positive feedback loop of lncRNA PVT1/miR-214/p53 possibly [20]. LncRNA RP11-89 facilitates tumorigenesis and ferroptosis resistance through PROM2-activated iron export by sponging miR-129-5p in bladder cancer [21]. In addition, long non-coding RNA NEAT1 promotes ferroptosis by modulating the miR-362-3p/MIOX axis as a ceRNA (competing endogenous RNA) [22]. Although ferroptosis is a key factor affecting the occurrence and development of tumors, although the role of ferroptosis in EC remains to be elucidated.

TMEM44-AS1 is a novel long non-coding RNA whose molecular biological functions has only recently begun to be investigate. The current study found that TMEM44-AS1 is up-regulated in a variety of tumors and can act as a proto-oncogene. For example, the super-enhancer-associated TMEM44-AS1 exacerbates glioma progression by forming a positive feedback loop with myc [23]. TMEM44-AS1 can sponge miR-2355-5p, leading to upregulation of PPP1R13L expression and inhibition of the P53 pathway to synergistically reverse 5-FU resistance in gastric cancer [24]. However, whether TMEM44-AS1 can regulate ferroptosis in EC has not been reported and the mechanism needs to be further investigated.

In this study, we analyzed the lncRNAs associated with ferroptosis in ESCC through bioinformatics analysis. A series of functional studies have shown that TMEM44-AS1 significantly induces the proliferation, migration, and invasion of ESCC cells in vitro and in vivo. Mechanistically, TMEM44-AS1 interacts with the m6A reader protein IGF2BP2 to induce increased stability of the target gene GPX4, thereby regulating the progression of ferroptosis in ESCC. In summary, our study proposes a promising clinical indicator and therapeutic target for the pathogenesis and treatment of ESCC.

## Results

### Expression and characterization of TMEM44-AS1 in ESCC tissues and cell lines

**Fig. 1:**
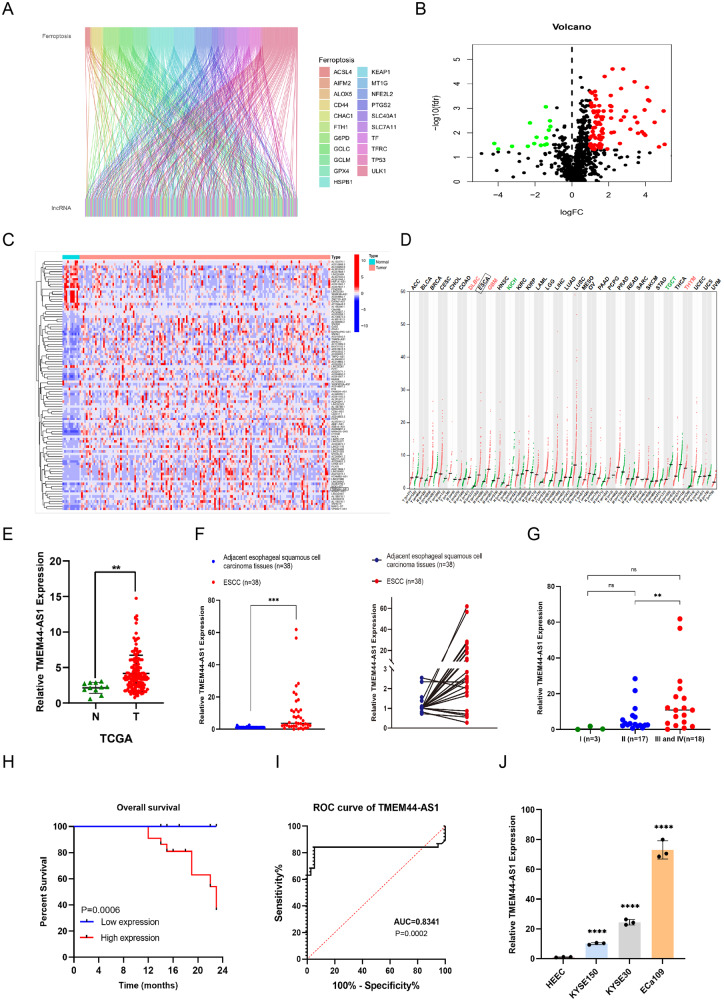
Expression and validation of TMEM44-AS1 in ESCC tissues and cells. **A** Enrichment analysis of lncRNA associated with ESCC and key genes of ferroptosis. **B** Volcano plot of differently expression ferroptosis-related lncRNAs. **C** Heatmap analysis of lncRNAs associated with ferroptosis with significant difference in ESCC. **D** The expression of TMEM44-AS1 in various cancers. **E** Expression of TMEM44-AS1 in ESCC in TCGA Database (fold change: 4.8847, *p* value: 0.0014). **F** Verify the expression of TMEM44-AS1 in ESCC tissue by qRT-PCR. **G** Correlation between TNM grading and TMEM44-AS1 expression displayed by qRT-PCR expression **H** Display the prognosis of TMEM44-AS1 in the K-M survival curve. **I** Display the diagnostic value of TMEM44-AS1 in the ROC curve. **J** Verification of TMEM44-AS1 expression in normal esophageal epithelial cells (HEEC) and three esophageal cancer cell lines (KYSE150, KYSE30, ECa109). *p < 0.05, **p < 0.01, ***p < 0.001, ****p < 0.0001.

Through bioinformatics analysis, we conducted co-expression analysis of lncRNA and key genes related to EC (Fig. 1A). Next, we obtained 100 lncRNAs that were abnormally expressed in EC through differential analysis (Fig. 1B, C). Then, we selected 15 ferroptosis-related lncRNAs significantly for qRT-PCR detection in EC cell lines. We found significant differences in the expression of LINC01116, DLEU2, LINC02381, SNGH19, and TMEM44-AS1 in EC cell lines. Subsequently, we performed qRT-PCR detection on these five lncRNAs in EC tissue. The results showed that TMEM44-AS1 was significantly overexpressed in both EC cell lines and EC tissues. (Supplemental Material: Fig. 1) Therefore, we preliminary selected TMEM44-AS1 as our target lncRNA and predicted the high expression level of TMEM44-AS1 in EC through the TCGA database (Fig. 1D, E). To further validate our hypothesis, we performed qRT-PCR validation on RNA extracted from 38 pairs of adjacent and EC tissues to verify the expression level of TMEM44-AS1 in EC (Fig. 1F). The correlation between the clinical pathological characteristics of EC patients and the expression level of TMEM44-AS1 is shown in Table 1. qRT-PCR results showed a close correlation between the expression of TMEM44-AS1 and TNM staging (Fig. 1G). The high expression of TMEM44-AS1 is significantly correlated with poor overall survival (OS), suggesting that high expression of TMEM44-AS1 is associated with poor prognosis (Fig. 1H). Similarly, the Receiver operating characteristic (ROC) was drawn according to the expression of TMEM44-AS1 in ESCC and precancerous tissues. The area under the curve (AUC) was 0.8341, indicating that the expression of TMEM44-AS1 in tissues is of high value for the diagnosis of ESCC (Fig. 1I). Subsequently, we validated the expression of TMEM44-AS1 in HEEC and EC cell lines (KYSE150, KYSE30, ECa109) (Fig. 1J). After verification, we preliminarily found that the expression of TMEM44-AS1 is upregulated in EC cells and tissues, with significant differences. This indicates that TMEM44-AS1 may regulate the progression of EC and is closely related to ferroptosis.

**Table 1:** Relationship between TMEM44-AS1 expression and clinicopathological characteristics in ESCC patients.

TMEM44-AS1 expression	*n*	Low	High	*P* value
Parameter				
All cases	38	16	22	
Age (year)	38			0.9950
<60	12	7	5	
≥60	26	11	15	
Sex	38			0.2793
Male	23	9	14	
Female	15	7	8	
TNM stage	38			*0.0271
I–II	20	12	8	
III–IV	18	4	14	

*TNM stage* tumor-node-metastasis stage.

*P* < 0.05 was considered as statistically significant.

*P < 0.05.

### TMEM44-AS1 induces ESCC cell malignant behaviors in vitro

**Fig. 2:**
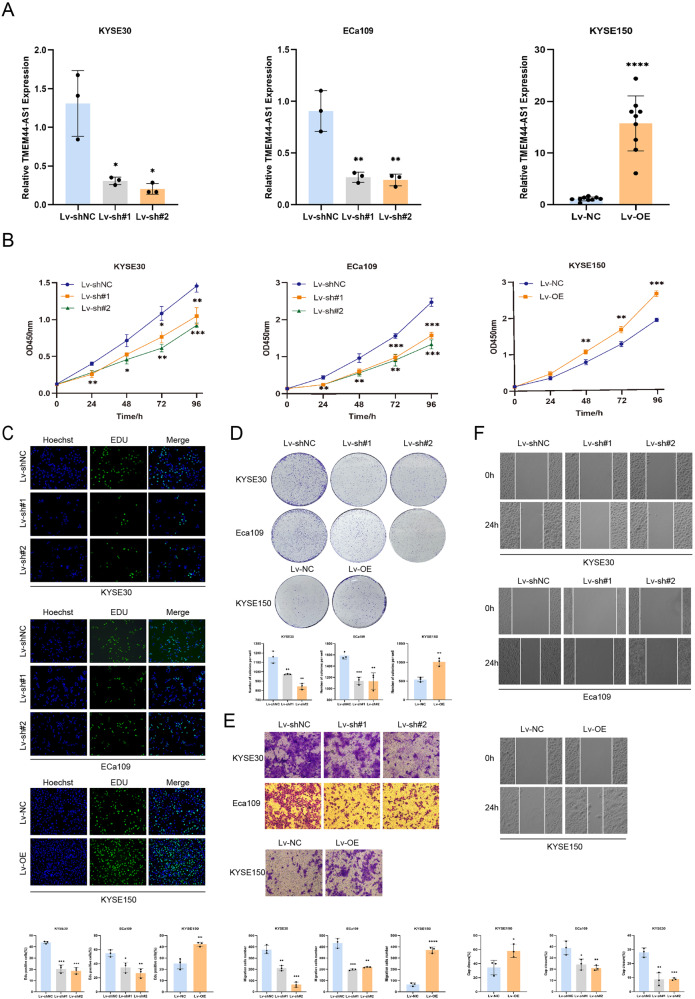
TMEM44-AS1 knockdown inhibits ESCC progression, while overexpression promotes ESCC progression. **A** Two types of lentivirus TMEM44-AS1-shRNA knockdown sequences were transfected with KYSE30 and ECa109, respectively. KYSE150 was transfected with lentivirus overexpression sequences, and the knockdown and overexpression efficiency were verified by qRT-PCR. **B** Evaluate the cell viability of KYSE30, ECa109, and KYSE150 cells through CCK-8 assay. **C** Detection of changes in cell proliferation after knockdown and overexpression of TMEM44-AS1 using EDU assay (amplification)×. (Scale: 100 μm). **D** Determine the changes in cell proliferation ability after knockdown and overexpression of TMEM44-AS1 through clone formation experiments. **E** Evaluate the migration and invasion ability of cells after knockdown and overexpression of TMEM44-A1 using transwell assay. **F** Evaluate the changes in cell migration ability after knockdown and overexpression of TMEM44-AS1 using wound healing assay. *p < 0.05, **p < 0.01, ***p < 0.001, ****p < 0.0001.

To investigate the biological function of TMEM44-AS1 in EC cells, we interfered with endogenous TMEM44-AS1 expression by transfecting a lentivirus with TMEM44-AS1-specific shRNA and a TMEM44-AS1 overexpression plasmid. After successful transfection, the knockdown and overexpression efficiencies were verified by qRT-PCR assays, and we found that the expression levels of TMEM44-AS1 were significantly reduced after knockdown in KYSE30 and EC109 cell lines and significantly increased after overexpression in KYSE150 cell line. The differences were statistically significant (Fig. 2A).

The CCK-8 assay showed that TMEM44-AS1 silencing resulted in a significant reduction in the absorbance at 450 nm of the EC cell lines KYSE30 and EC109 (Fig. 2B). Similarly, Edu analysis showed that the number of Edu-positive cells was significantly reduced in the TMEM44-AS1 silencing group compared to the negative control group for both KYSE30 and EC109 cell lines (Fig. 2C). Colony formation assays showed that KYSE30 and EC109 cells proliferated significantly slower after TMEM44-AS1 compared to the control group (Fig. 2D). Conversely, after TMEM44-AS1 overexpression, the KYSE150 cell line showed a significant increase in OD450, a significant increase in EDU positivity and a significantly higher rate of colony formation. These results suggest that lncRNA TMEM44-AS1 enhances the proliferation of EC cells, whereas the proliferation of EC cells was significantly inhibited when TMEM44-AS1 was silenced.

In addition, transwell assays demonstrated that KYSE30, EC109 cell invasion capacity was reduced after TMEM44-AS1 silencing (Fig. 2E). Wound healing assays demonstrated that the migration ability of KYSE30, EC109 cell lines was diminished after TMEM44-AS1 silencing compared to negative controls (Fig. 2F). In contrast, KYSE150 cell lines overexpressing TMEM44-AS1 had increased migratory and invasive capacity compared to the control group. These results suggest that TMEM44-AS1 can enhance the migration and invasion ability of EC cells, and that the migration and invasion levels of EC cells were reduced after silencing of TMEM44-AS1.

### TMEM44-AS1 affected cell cycle and diminished cell apoptosis of ESCC

**Fig. 3:**
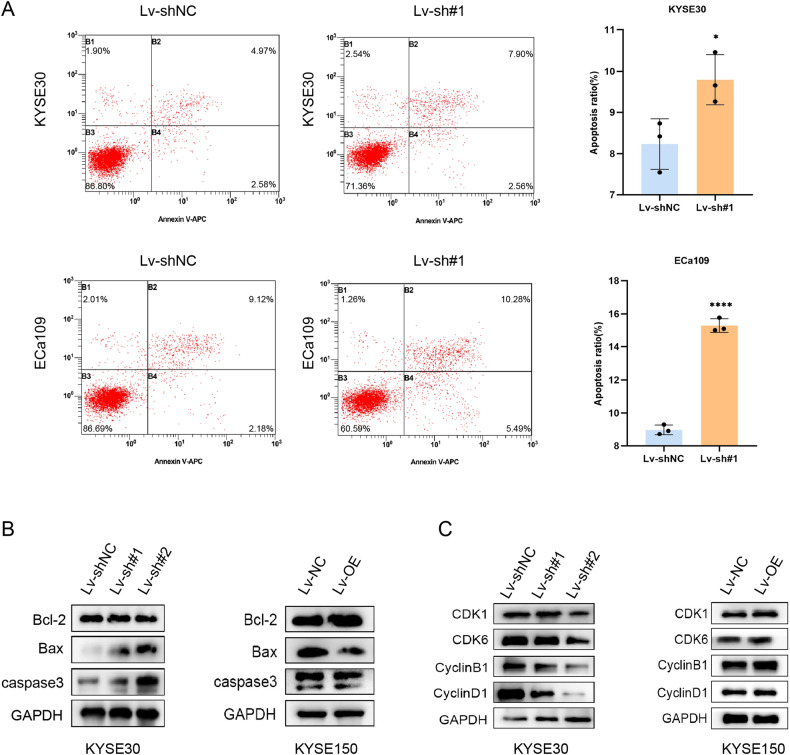
TMEM44-AS1 induces ESCC cell apoptosis and participates in regulating cell cycle. **A** Detect the changes in cell apoptosis after knocking down KYSE30 and overexpressing KYSE150 using flow cytometry. **B** Western blot was used to detect changes in key apoptotic proteins (bcl-2, bax, and caspase-3) after knocking down KYSE30 and overexpressing KYSE150. **C** Western blot was used to detect changes in key proteins (CDK1, CDK6, cyclinB1, and cyclinD1) in the cell cycle after knocking down KYSE30 and overexpressing KYSE150. *p < 0.05, **p < 0.01, ***p < 0.001, ****p < 0.0001.

The effect of TMEM44-AS1 on the anti-apoptotic and cell cycle capacity of EC cells was analyzed by flow cytometry and western blot. Flow cytometry analysis revealed a significant increase in the number of early and late apoptotic cells following knockdown of TMEM44-AS1 (Fig. 3A). Western blotting of intracellular apoptotic proteins showed that knockdown of TMEM44-AS1 resulted in a decrease in the anti-apoptotic protein bcl-2 and an increase in capase3 and bax levels. In contrast, overexpression of TMEM44-AS1 resulted in increased levels of bcl-2 and decreased levels of capase3 and bax. These results suggest that TMEM44-AS1 plays an anti-apoptotic role in EC (Fig. 3B).

The levels of cell cycle-related proteins (CDK1, CDK6, cyclin B1, and cyclinD1) were significantly reduced in KYSE30 and EC109 cells after TMEM44-AS1 silencing, whereas cell cycle-related proteins were elevated in KYSE150 cells overexpressing TMEM44-AS1. This suggests that knockdown of TMEM44-AS1 blocked the cycle of tumor cells. These results suggest that TMEM44-AS1 has a positive regulatory effect on the cell cycle (Fig. 3C).

### TMEM44-AS1 participates in regulating ESCC ferroptosis and positively regulates GPX4

**Fig. 4:**
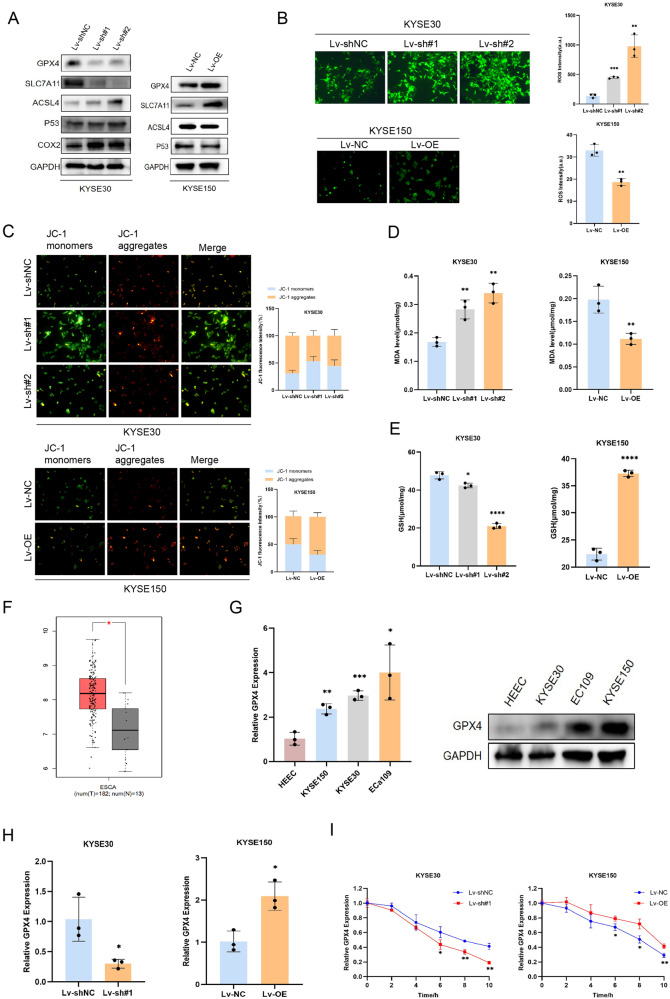
TMEM44-AS1 inhibits ESCC ferroptosis and is positively correlated with GPX4. **A** Western blot was used to detect the changes of key proteins of ferroptosis after knockdown and overexpression of TMEM44-AS1. **B** After the addition of ferroptosis inducer erastin, the levels of ROS in ESCC cells after knockdown and overexpression of TMEM44-AS1 were detected through ROS experiments. **C** Detection of mitochondrial membrane potential levels in ESCC cells with knockdown and overexpression of TMEM44-AS1 after the addition of ferroptosis inducer erastin through JC-1 experiment. **D** Detect the lipid oxidation levels of ESCC cells after knocking down and overexpression of TMEM44-AS1 with the addition of ferroptosis inducer eratin through MDA experiments. **E** Detection of glutathione levels in ESCC cells after knockdown and overexpression of TMEM44-AS1 after the addition of ferroptosis inducer erastin through GSH experiments. **F** TCGA database predicts the expression of GPX4 in ESCC. **G** Verifying the expression of GPX4 in HEEC, KYSE150, KYSE30, and ECa109 through qRT-PCR and Western blot analysis. **H** Verify the expression level of GPX4 after knocking down and overexpressing TMEM44-AS1 through qRT-PCR. **I** The change of GPX4 mRNA level after knockdown and overexpression of TMEM44-AS1 was verified by Dactinomycin D experiment. *p < 0.05, **p < 0.01, ***p < 0.001, ****p < 0.0001.

Predicting through TCGA database, we found that TMEM44-AS1 was associated with ferroptosis, which was validated by a series of ferroptosis experiments. First of all, we verified the expression of key proteins of ferroptosis by Western blot experiment on the protein extracted from the EC cell line (KYSE30, ECa109, KYSE150) constructed by Lentivirus. The results showed that after knocking down TMEM44-AS1, the protein expression level of ferroptosis-promoting genes increased, while the protein expression level of ferroptosis inhibiting genes decreased (Fig. 4A). Next, we added the ferroptosis inducer erastin to treat ESCC cells, and found that knocking down TMEM44-AS1 resulted in increased levels of ferroptosis in ESCC cells, including increased levels of lipid ROS, reduced levels of cellular mitochondrial membrane potential, increased levels of MDA and depletion of GSH (Fig. 4B–E). This suggests that TMEM44-AS1 is closely associated with ferroptosis and that knockdown of TMEM44-AS1 leads to increased levels of ferroptosis.

On the contrary, the situation is completely opposite after overexpression of TMEM44-AS1. This indicates that overexpression of TMEM44-AS1 can inhibit ferroptosis, thereby promoting tumor development to a certain extent. In summary, these experiments indicate that TMEM44-AS1 can regulate the onset of cellular ferroptosis, and knockdown of TMEM44-AS can promote ferroptosis. On the contrary, overexpression of TMEM44-AS1 can inhibit ferroptosis.

TMEM44-AS1 is closely related to various ferroptosis key genes, however, we found that the correlation between TMEM44-AS1 and GPX4 is most significant. By using the GEPIA database. We found that the expression of GPX4 was significantly higher in ESCA samples than in normal samples (Fig. 4F). Next, we validated the expression levels of GPX4 in HEEC and EC cell lines (KYSE150, KYSE30 and ECa109) using qRT-PCR and Western blotting (Fig. 4G). We then examined the expression of GPX4 in TMEM44-AS1 knockdown and overexpression cell lines by qRT-PCR, and confirmed that GPX4 was positively correlated with TMEM44-AS1 expression (Fig. 4H). Therefore, GPX4 was chosen for further investigation. The effect of TMEM44-AS1 on GPX4 mRNA was also observed using the Actinomycin D assay. The relative expression and stability of GPX4 was reduced in the TMEM44-AS1 knockdown cell line. However, the situation was reversed when TMEM44-AS1 was overexpressed (Fig. 4I). The above results suggest that TMEM44-AS1 is positively correlated with GPX4 expression and regulates ferroptosis occurrence through GPX4 expression levels.

### TMEM44-AS1 enhances the stability of GPX4 mRNA by interacting with IGF2BP2

**Fig. 5:**
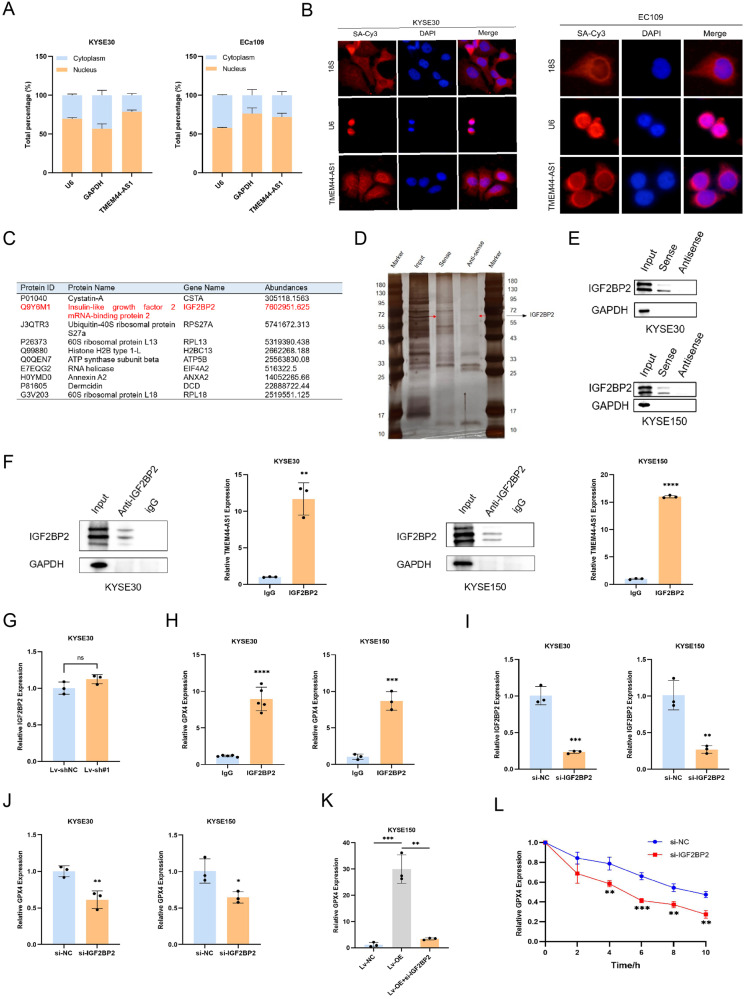
TMEM44-AS1 regulates the stability of GPX4 mRNA by interacting with IGF2BP2. **A** Detection of the distribution and localization of TMEM44-AS1 in cells through nucleocytoplasmic separation experiments. **B** FISH determination of TMEM44-AS1 in KESE30 and ECa109 cells. The nucleus was stained with DAPI, and the cytoplasm was stained with 18S. **C** Mass spectrum results showed IGF2BP2 can bind to TMEM44-AS1 sense group. **D** Verify the interaction between TMEM44-AS1 and IGF2BP2 through RNA pull-down experiments and silver staining. **E** Verify the interaction between TMEM44-AS1 and IGF2BP2 through Western blot experiments. **F** Verify the interaction between TMEM44-AS1 and IGF2BP2 through RIP experiments. **G** After knocking down TMEM44-AS1, there was no significant change in the expression level of IGF2BP2. **H** Verify the interaction between IGF2BP2 and GPX4 through RIP experiments. **I** Verify the knockdown efficiency of IGF2BP2 through qRT-PCR. **J** Knockdown the expression level of GPX4 after knocking down IGF2BP2. **K** The expression level of GPX4 after overexpression of TMEM44-AS1 and treatment with overexpression of TMEM44-AS1 combined with si-IGF2BP2. **L** The stability of GPX4 mRNA level after knocking down IGF2BP2 was verified by Dactinomycin D experiment. *p < 0.05, **p < 0.01, ***p < 0.001, ****p < 0.0001.

To further investigate the mechanism of TMEM44-AS1 regulation of GPX4. Verify the distribution of TMEM44-AS1 in ESCC cells through nuclear cytoplasmic separation experiments (Fig. 5A). The cellular localization of TMEM44-AS1 in ESCC cells was investigated using RNA fluorescence in situ hybridization (FISH) (Fig. 5B). The results showed that TMEM44-AS1 was mainly localized in the cytoplasm of ESCC cells, and we hypothesized that TMEM44-AS1 might function in ESCC cells through post-transcriptional regulation. It was suggested that lncRNA may interact with RNA-binding proteins and regulate their downstream target genes. IGF2BP2, a classical RBP (RNA Binding Protein), has been shown in several studies to be involved in regulating mRNA stability and thus influencing tumor progression. The mass spectrometry results indicate that IGF2BP2 can bind to TMEM44-AS1 (Fig. 5C). Then, we designed a full-length biotin-labeled TMEM44-AS1 sense and antisense sequence probe, and after RNA pull-down and silver staining, specific bands were found (Fig. 5D). Western blotting was then performed to discover the interaction of TMEM44-AS1 with IGF2BP2, and the results showed that TMEM44-AS1 but not antisense captured IGF2BP2 (Fig. 5E). Next, the binding of IGF2BP2 to TMEM44-AS1 was confirmed by RIP assay. The immunoprecipitated RNA was analyzed by qPCR and the efficiency of immunoprecipitation was verified by protein blotting. Anti-IGF2BP2 enriched more TMEM44-AS1 RNA than anti-IgG (Fig. 5F). However, there was no significant change in the expression level of IGF2BP2 after knocking down TMEM44-AS1 (Fig. 5G). These results confirmed the interaction between IGF2BP2 protein and TMEM44-AS1 RNA.

IGF2BP2 has been reported as a RNA-binding protein associated with mRNA stability, and we hypothesize that TMEM44-AS1 stabilizes GPX4 mRNA by interacting with IGF2BP2. Subsequently, RIP assays were performed to verify the interaction between IGF2BP2 and GPX4. GPX4 mRNA was strongly enriched in the anti-IGF2BP2 group compared to the anti-IgG group (Fig. 5H). Next, we knocked down IGF2BP2 and detected knockdown efficiency through qRT-PCR (Fig. 5I). After knocking down IGF2BP2, the expression level of GPX4 significantly decreased (Fig. 5J). After overexpression of TMEM44-AS1, GPX4 showed high expression and could be rescued by the combination of si-IGF2BP2 (Fig. 5K). Subsequently, after treatment with actinomycin D, the mRNA stability of GPX4 decreased at the planned time point, indicating that TMEM44-AS1 may interact with IGF2BP2 in ESCC to promote the stability of GPX4 mRNA (Fig. 5L).

### TMEM44-AS1 affects ESCC cell proliferation, invasion, and migration by regulating GPX4

**Fig. 6:**
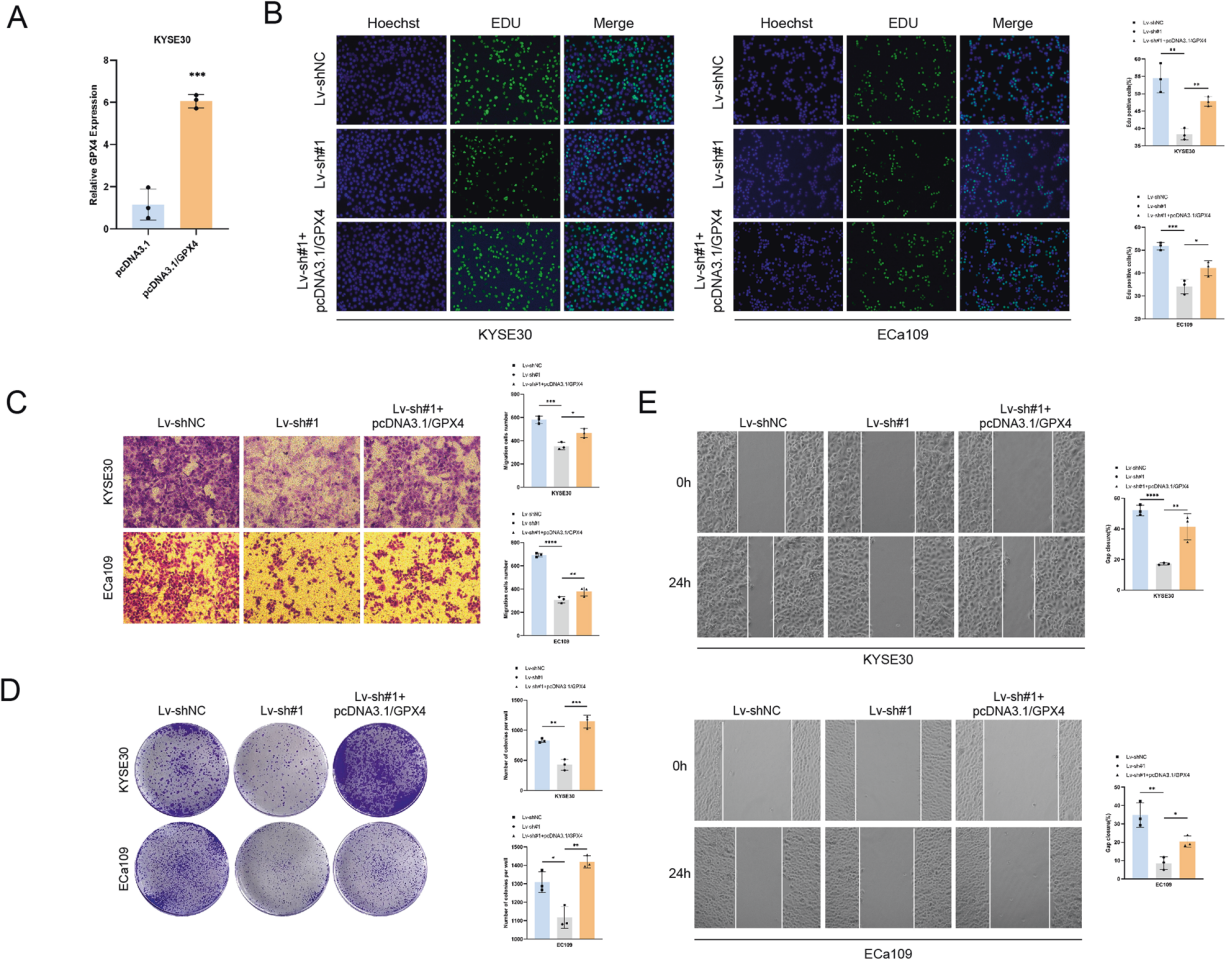
Overexpression of GPX4 can salvage the progression of ESCC regulated by TMEM44-AS1. **A** Verifying the efficiency of overexpressing GPX4 through qRT-PCR. **B** EDU experiment verifies that overexpression of GPX4 can restore the inhibition of ESCC proliferation caused by knocking down TMEM44-AS1. **C** Transwell experiment verifies that overexpression of GPX4 can restore the inhibition of ESCC invasion caused by knocking down TMEM44-AS1. **D** Clone formation experiment verifies that overexpression of GPX4 can restore the inhibition of ESCC growth caused by knocking down TMEM44-AS1. **E** Wound healing experiment verifies that overexpression of GPX4 can restore the inhibition of ESCC migration caused by knocking down TMEM44-AS1. *p < 0.05, **p < 0.01, ***p < 0.001, ****p < 0.0001.

TMEM44-AS1 plays a role in GPX4 mRNA stability, and we wanted to further investigate whether TMEM44-AS1 is involved in the development of EC through GPX4. First, we transfected the GPX4 overexpression plasmid in KYSE30 and examined the overexpression efficiency by qPCR (Fig. 6A). Then we performed reversion experiments on ESCC cell proliferation, migration, invasion, and ferroptosis. EDU and clone formation assays showed that GPX4 overexpression rescued the diminished proliferative capacity of ESCC cells caused by TMEM44-AS1 knockdown (Fig. 6B, D). Transwell and scratch assays showed that GPX4 overexpression rescued the diminished invasive and migratory capacity of ESCC cells caused by TMEM44-AS1 knockdown (Fig. 6C, E). Taken together, the results suggest that TMEM44-AS1 can promote the function of ESCC cells in a GPX4-dependent manner thereby affecting EC progression.

### TMEM44-AS1 affects ESCC cell ferroptosis by regulating GPX4

**Fig. 7:**
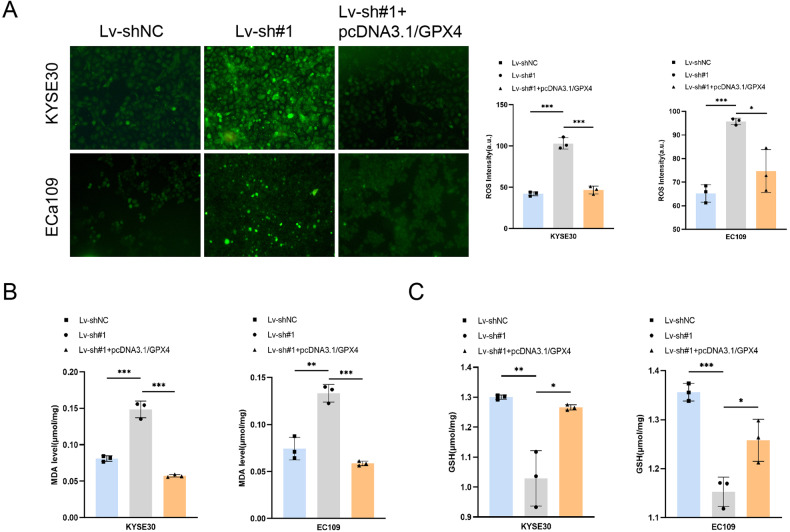
Overexpression of GPX4 reverts to TMEM44-AS1 mediated ESCC ferroptosis. **A** ROS experiments have confirmed that the overexpression of GPX4 can reverse the increase in ROS levels after knocking down TMEM44-AS1 by adding the ferroptosis inducer erastin. **B** MDA experiments have confirmed that the overexpression of GPX4 can reverse the increase in MDA levels after knocking down TMEM44-AS1 by adding the ferroptosis inducer erastin. **C** GSH experiment confirmed that the overexpression of GPX4 after the addition of ferroptosis inducer erastin can reverse the increase in glutathione levels in cells after knocking down TMEM44-AS1. *p < 0.05, **p < 0.01, ***p < 0.001, ****p < 0.0001.

TMEM44-AS1 can affect cell ferroptosis by regulating GPX4. Adding ferroptosis inducer erastin to ESCC cells resulted in an increase in ROS levels after knocking down TMEM44-AS1. When combined with overexpression of GPX4, ROS levels were reversed (Fig. 7A). Similarly, knocking down TMEM44-AS1 resulted in an increase in MDA levels, while combined overexpression of GPX4 resulted in a significant decrease in MDA levels (Fig. 7B). Knocking down TMEM44-AS1 resulted in an increase in GSH levels, while co-overexpression of GPX4 resulted in a complete reversal of the situation (Fig. 7C). In conclusion, the results show that TMEM44-AS1 can inhibit ferroptosis of ESCC cells in a GPX4 dependent manner, thereby promoting the malignant progression of ESCC.

### TMEM44-AS1 plays oncogenic effects in vivo

**Fig. 8:**
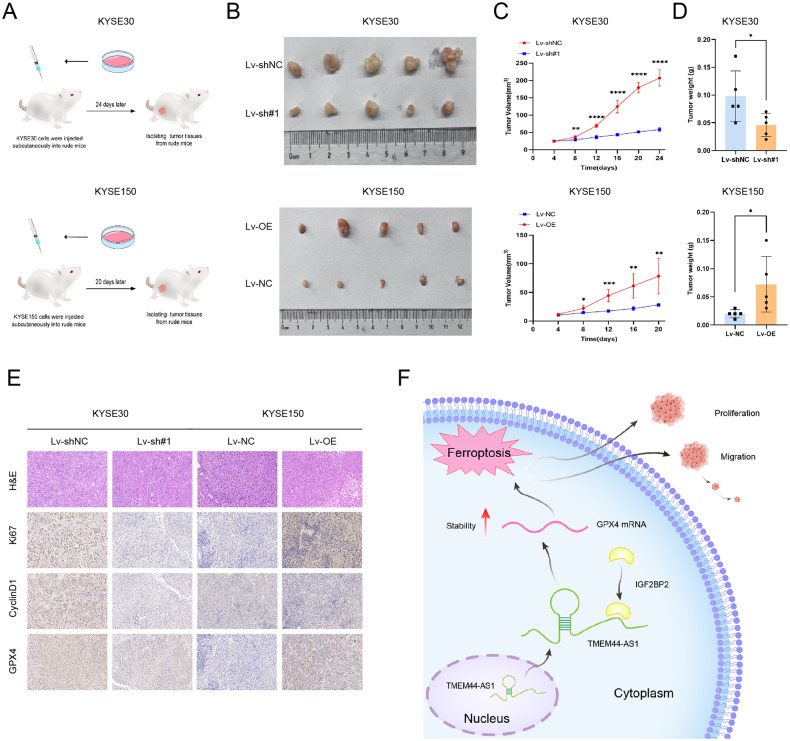
TMEM44-AS1 accelerates the occurrence of tumors in vivo. **A** The process of constructing a subcutaneous tumor model in nude mice. **B** Image showing knockdown and overexpression of TMEM44-AS1 subcutaneous tumor. **C** The tumor volume of the indicator group is measured every 4 days. **D** Quantify the weight of subcutaneous tumors. **E** The H&E staining and IHC staining of xenograft tumors include Ki-67, cyclinD1, and GPX4. **F** Schematic illustration of the biological role of TMEM44-AS1/IGF2BP2/GPX4 axis in modulating ESCC malignant progression. *p < 0.05, **p < 0.01, ***p < 0.001, ****p < 0.0001.

To assess the in vivo biological role of TMEM44-AS1 in esophageal carcinogenesis, we constructed a subcutaneous xenograft model and inoculated nude mice with sh-TMEM44-AS1 after stable transfection of KYSE30 cells and KYSE150 (Fig. 8A). Compared to controls, TMEM44-AS1 knockdown resulted in significantly lower tumor volume and weight and reduced tumor growth rate (Fig. 8B–D). H&E staining and immunohistochemistry showed that knockdown tumor tissue had decreased ki67, decreased cyclinD1, and decreased GPX4 expression (Fig. 8E). Interestingly, after overexpression of TMEM44-AS1, the above results were completely consistent. These results suggest that TMEM44-AS1 is an oncogenic lncRNA that inhibits ferroptosis and promotes esophageal carcinogenesis in vivo.

## Discussion

In recent years, numerous studies have shown that lncRNAs play a key role in tumor progression. TMEM44-AS1 is a newly discovered lncRNA that has been found to play a role in gastric cancer and glioma. TMEM44-AS1 competitively binds to miR-2355-5p to upregulate PPP1R13L expression and repress the P53 pathway. Knockdown of TMEM44-AS1 also synergistically reversed 5-FU resistance in gastric cancer and enhanced the efficacy of 5-FU treatment in gastric cancer patients [24]. In gliomas, TMEM44-AS1 binds to SerpinB3 and activates myc and EGR1\IL-6 signaling. Myc transcription induces TMEM44-AS1 and binds directly to the promoter and super enhancer of TMEM44-AS1, thus forming a positive feedback loop with TMEM44AS and participating in the regulation of glioma development [23]. In this study, we report for the first time the oncogenic role of TMEM44-AS1 in ESCC. TMEM44-AS1 was highly expressed in ESCC cell lines and promoted tumor growth in vitro and in vivo. This suggests that TMEM44-AS1 is positively associated with ESCC progression.

RNA binding proteins can mediate the post transcriptional regulation of lncRNA [25]. IGF2BP2, a member of the IGF2BP family, can mediate post-transcriptional fine regulation of the expression of genes related to tumor cell proliferation, survival, chemoresistance, and metastasis. The expression of IGF2BP family members was found to be associated with overall poor prognosis and metastasis in various human cancers [26]. Emerging evidence suggests that IGF2BP2 is an N6-methyladenosine (m6A) reader that is involved in cancer progression by interacting with lncRNAs [27]. Hou et al. found that LINC00460 increased the stability of HMGA1 mRNA by interacting with IGF2BP2 and DHX9 in an m6A modification-dependent manner, enhancing HMGA1 expression to promote proliferation and metastasis of colorectal cancer cancers [28]. Similarly, LINC01559 can enhance the stability of ZEB1 mRNA by recruiting IGF2BP2, accelerate the proliferation and migration of gastric cancer cells, and promote the malignant development of gastric cancer [29]. Linc01305 enhances the mRNA stability of HTR3A by interacting with IGF2BP2 and IGF2BP3, promoting the proliferation and migration of ESCC [30]. Liu et al. found that lncRNA PACERR binds to IGF2BP2 and enhances the stability of KLF12 and c-myc in the cytoplasm in an m6A-dependent manner, thereby mediating the proliferation, invasion, and migration of pancreatic ductal adenocarcinoma cells and regulating cancer progression [31]. In our study, we discovered a new mechanism of IGF2BP2 in ESCC. Specifically, IGF2BP2 can interact with TMEM44-AS1, enhancing the stability of GPX4 mRNA, increasing the expression level of GPX4, thereby inhibiting ferroptosis in ESCC cells. However, how TMEM44-AS1 acts as a molecular scaffold between IGF2BP2 and GPX4 needs to be further explored.

Herein, we also found a significant upregulation of GPX4 expression in ESCC cell lines, and a positive correlation between GPX4 and TMEM44-AS1. GPX4 is a key regulatory factor for ferroptosis, inhibiting COX and lipoxygenase (LOX) activity and cell ferroptosis by inhibiting cell lipid peroxidation [32, 33]. Li et al. found that CST1 reduces ubiquitination modification of GPX4 by recruiting OTUB1, improves GPX4 protein stability, and reduces intracellular ROS, thereby inhibiting ferroptosis and promoting the development and metastasis of gastric cancer [34]. Similarly, Lei et al. found that linc00976 sponge miR-3202 upregulates the expression level of GPX4, which promotes the progression and metastasis of cholangiocarcinoma and inhibits ferroptosis [35].

In our study, TMEM44-AS1 can upregulate the expression level of GPX4, we conducted a series of rescue experiments in vitro and found that overexpression of GPX4 can restore ferroptosis caused by knocking down TMEM44-AS1, promoting the proliferation, migration, and invasion of ESCC cells. However, further research is needed to investigate the mechanism by which TMEM44-AS1 stabilizes GPX4 mRNA stability through its interaction with IGF2BP2. In summary, we have identified for the first time the biological function and specific mechanisms of TMEM44-AS1 in the progression of ESCC (Fig. 8F). Our study found that the TMEM44-AS1-IGF2BP2-GPX4 axis is involved in the pathogenesis and progression of ESCC, providing new diagnostic biomarkers and therapeutic targets for ESCC patients. Overall, our findings reveal a novel signaling pathway that may help develop new therapeutic strategies for ESCC patients.

## Materials and methods

### Bioinformatics mechanism exploration

Download the complete transcriptome data of EC from The Cancer Genome Atlas (TCGA) database. Data included a total of 182 tumor samples and 11 normal samples. A total of 259 ferroptosis-related genes contained driver, suppressor, and marker that were extracted from the FerrDb database. Then, we used Pearson correlation analysis to calculate the correlation between lncRNAs and ferroptosis-related genes, the filter standard correlation coefficient and *P*-value were set to 0.4 and 0.05, respectively. Differential expression ferroptosis-related lncRNAs was obtained through the R limma package and was used to draw the heatmap and volcano plot, the screening criteria were |logFC| value > 1, *P* < 0.05. The relationship between 21 ferroptosis markers and ferroptosis-related lncRNAs was obtained through R limma, dplyr, ggalluvial, and ggplot2 packages and was used to draw the Sankey map.

### Clinical tissue specimens

The fresh tumor tissue and corresponding adjacent normal esophageal epithelium used in this experiment were taken from EC patients admitted to Anyang Cancer Hospital. All subjects were diagnosed by the Pathology Department of Anyang Cancer Hospital and did not undergo surgery, radiation therapy, or chemotherapy before surgery. After surgical resection, all samples were immediately stored in liquid nitrogen at −80 °C until RNA was extracted. This experiment was approved by the Biomedical Ethics Committee of Anyang Cancer Hospital (2022WZ12K01).

### Cell culture

The human normal esophageal epithelial cell line HEEC and the human ESCC cell lines (KYSE30, KYSE150, ECa109) purchased from the Institute of Cells, Shanghai Academy of Life Sciences. Each cell line was cultured in RPMI-1640 medium (Yuanpei Biotechnology Co., Ltd, Shanghai) supplemented with 10% fetal bovine serum (Cell- Box Biological products Trading Co., Ltd) and 1% penicillin-streptomycin (Solarbio, China). Each EC cell line was stored at 37 °C in a 5% nitrogen dioxide incubator.

### Lentivirus production, plasmid construction, and infection

TMEM44-AS1 was knockdown or overexpression by using the TMEM44-AS1 shRNA or full- length sequences- packaged lentivirus. The lentiviral vector systems and overexpression plasmids were all purchased from GeneChem (Shanghai, China). The overexpression plasmid of GPX4 was purchased from GeneChem (Shanghai, China). Puromycin was used to select the transfected cells for 1 week. Small interfering RNAs targeting (si-IGF2BP2) and their relative control (si-NC) were purchased from (Sangon Bioengineering Co., Ltd, Shanghai). Lipofectamine 2000 (Invitrogen, USA) was used to terminate all transfections. All procedures were carried out according to the manufacturer’s instructions.

### RNA extraction and quantitative real‑time polymerase chain reaction (qRT-PCR)

Total RNA of cells was extracted by TRIzol reagent (Takara, Kyoto, Japan). The complementary DNA was reverse transcribed buy PrimeScript™ RT rea-gent Kit (Takara, Kyoto, Japan). Quantitative real-time PCR (qPCR) was applied to detect expression quantity by using TB green Premix Ex Taq™ II kit (Takara, Kyoto, Japan) on LightCycler480 system (Roche, Swiss). Actin served as an internal reference. Relative expression was calculated using the 2 − ΔΔCT method. Each experiment was replicated for three times.

### Cell counting kit-8

The viability of the cells was measured using Cell Counting Kit - 8 reagent (CCK-8) (Dojindo Laboratories, Kumamoto, Japan). The number of cells per well in 96 well plates was 2000, and 10 µL CCK8 reagent was added to 100 µL medium per well. Incubate at 37 °C and 5% carbon dioxide for 2 h in dark, and measure the absorbance at 450 nm at 0, 24, 48, 72, and 96 h respectively.

### EDU assay

100 μl of cell suspension (2 × 103 cells) was plated in 96 well plates and incubated overnight. 50 μM EdU reagent (Beyotime technology, Shanghai) was added and incubated for 2 h. Cells were then fixed with formaldehyde, and the nucleus was Hoechst stained. Finally, observed and photographed with fluorescence microscope.

### Transwell migration assay

The capacity for cell migration and invasion was measured by the Transwell method. Briefly, the transfected cells were inoculated with 5 × 104 cells in a 200 µL serum free medium suspension. Add 600 µL complete culture medium without penicillin-streptomycin in the bottom chamber. Cultured at 37 °C in a 15% carbon dioxide incubator for 48 h. After removal of the top layer of cells, the chambers were fixed in 4% paraformaldehyde fixative at room temperature for 30 min and then stained with crystal violet, and three fields were randomly selected under the microscope to count the cell migration.

### Wound healing assays

Inoculate the transfected cells into a 6-well plate, and wait until the fusion degree reaches 90%. Sweep the wound straightly with a 10 µL sterile pipette tip, and rinse the wound with PBS for three times. Cells were grown in serum-free medium for 24 h, and the images of 0 and 24 h were recorded. The experiment was repeated three times. Finally, the wound area was analyzed by ImageJ (NIH, USA).

### Colony formation

The ESCC cells were seeded in a six-well plate with a density of 2000 cells per well. Then, cells were grown for 6 days at 37 °C in a 5% carbon dioxide incubator and fixed with 4% paraformaldehyde for one-half hour. Lastly, cells were stained with 0.5% crystal violet.

### Apoptosis detection assay

The degree of apoptosis following transfection was assessed by Annexin V-FITC /PI staining (BD Biosciences, San Jose, CA, USA) and flow cytometry (FACScan, BD Biosciences). Transfected cells were subjected to trypsin digestion, washed three times with PBS, collected in a flow tube, centrifuged at 1000 *g* for 5 min, and the supernatant was discarded. Use 50 μL binding buffer to resuspend the cells, and then add 5 μL Annexin-V and PI were incubated in dark for 15 min. Supplement binding buffer to 500 μL. Flow cytometry was used for detection.

### Protein extraction and western blotting

Cells were lysed on ice using RIPA (New cell & Molecular Biotech Co., Ltd). Protein concentration was detected using the BCA protein quantification kit (Beyotime, Shanghia, China), and the protein concentration was calculated as follows.

Protein samples were separated on 10% SDS-PAGE gels (Epizyme Biomedical Technology Co., Ltd) and then transferred to PVDF membranes. protein samples were analyzed by Western blotting. The membrane was then incubated with 5% nonfat milk for 1 h followed by incubation with primary antibodies overnight at 4 °C. HRP- conjugated secondary antibodies were then washed three times and the membranes were added for incubation for 1 h at room temperature. The blots were visualized with ECL kit (Epizyme Biomedical Technology Co., Ltd). The primary antibodies TMEM44-AS1, IGF2BP2, and GAPDH were bought from Proteintech (Wuhan, China). The following antibodies were used: anti-IGF2BP2 (11601–1-AP, Proteintech, China), anti-GPX4 (ab125066, Abcam, USA) GAPDH (60004-1-lg, Proteintech, China), COX2 (66351-1-lg, Proteintech, China), TP53 (60283-2-lg, Proteintech, China), SLC7A11 (26864-1-AP, Proteintech, China), anti-ACSL4 (ab155282, Abcam, USA), Tubulin (11224-1-AP, Proteintech, China), Caspase 3 (19677-1-AP, Proteintech, China), Bcl-2 (26593-1-AP, Proteintech, China), Bax (50599-2-lg, Proteintech, China), CDK1 (CY5176, Abways, China), CDK6 (CY5835, Abways, China), Cyclin B1 (CY5378, Abways, China), Cyclin D1 (CY5404, Abways, China).

### Lipid ROS assays

ESCC cells were seeded in six‐well cell culture plates and prepared for cell slides. Cells were treated with 8 mmol/L of N‐acetyl‐L‐cysteine (NAC) (Sigma‐Aldrich) or DMSO for 2 h. Then treated with 10 µM 2,7‐Dichlorodi ‐hydrofluorescein diacetate (Beyotime) for 20 min in the dark. The generation of ROS in cells was observed under fluorescence microscope.

### Lipid peroxidation assays

Collect ESCC cells from a six-well plate, the lipid peroxidation product MDA concentration in ESCC cell lysates was assessed using a Lipid Peroxidation MDA Assay Kit (S0131S, Beyotime, China) according to the manufacturer’s protocol. The total cell content was measured by a BCA kit (P0012, Beyotime). The obtained MDA absorbance was brought to the standard curve, and the corresponding MDA content was calculated.

### Glutathione assays

Collect ESCC cells from a six-well plate, GSH concentration in ESCC cell lysates was measured using a Glutathione Assay Kit (S0053, Beyotime) according to the manufacturer’s instructions. The total cell content was measured by a BCA kit (Beyotime). The obtained GSH absorbance was brought to the standard curve to obtain the corresponding GSH levels.

### Mitochondrial membrane potential assay

ESCC cells were seeded in six‐well cell culture plates and prepared for cell slides. Mitochondrial membrane potential level in ESCC cell lysates was measured using a Mitochondrial membrane potential assay kit with JC-1 (C2006, Beyotime) according to the manufacturer’s instructions. Changes in mitochondrial membrane potential were detected by taking photographs under a laser Confocal Microscope and shifting from red to green fluorescence.

### Fluorescencein situhybridization (FISH) assays

Cy3-labeled specific probes to TMEM44-AS1 and positive control probes (GenePharma, Shanghai, China) were designed and synthesized to detect the location of TMEM44-AS1 in KYSE30 cells. In short, after 30 min of pre hybridization at 37 °C, the cell slides were mixed with 2.5 μL-specific Cy3 labeled TMEM44-AS1 probe and positive control probe (20 μ M) Hybridize overnight at 37 °C and stain with DAPI. Photographs of the slide are taken using a laser scanning confocal microscope (Zeiss, Germany).

### RNA immunoprecipitation (RIP)

To verify the interaction of IGF2BP2 protein with TMEM44-AS1 and GPX4 RNA, an RIP assay was accomplished according to the manufacturer’s protocol of the RNA Immunoprecipitation Kit (Cat#P0102, Geneseed Biotech). RNAs and proteins were isolated and then detected by qPCR and western blot.

### RNA pull-down

Full-length biotin-labeled TMEM44-AS1 and antisense sequences probe were synthesized by Gemma Gene. Then RNA pull-down assays were performed according to the Magnetic RNA-Protein Pull- Down Kit manufacturer’s instructions. (Cat#20164, Thermo Scientific). Captured sense and antisense probe proteins were detected using mass spectrometry (APTBIO) and western blotting.

### Actinomycin D and RNA stability assay

KYSE30 cells were treated with 5 μg/mL actinomycin D at 0 h, 2 h, 4 h, 6 h, 8 h, 10 h respectively. Then cells were harvested to extract total RNA. The stability of TMEM44-AS1 and GPX4 mRNA was analyzed using qRT-PCR.

### Immunohistochemistry (IHC) and hematoxylin and eosin (H&E) staining

Paraffin sections were oven-dried, dewaxed in xylene, and then dipped in gradient eth-anol to allow for hydration. Samples were then washed with PBS and heated in boiling citrate buffer (pH 6.0) for 10 min to allow for antigens to be recovered. Sections were then incubated at room temperature with 3% H2O2 for 10 min and blocked with 5% normal goat serum at room temperature for 30 min followed by incubation with the anti-IGF2BP2 primary antibodies (Proteintech, China), anti-GPX4 (Proteintech, China), anti-Ki67 (Proteintech, China) at 4 °C overnight. After 2–3 rinses in PBS, the sections were incubated with biotinylated anti-IgG secondary antibody and horseradish peroxidase-labeled streptavidin was added to the slides and incubated for 15 min. The final immunoreactivity score of GPX4 was calculated as previously described. For H&E staining, the nucleus and cytoplasm were stained with hematoxylin and eosin, respectively. General histological examination was performed by standard procedures.

### Xenograft models

KYSE30 cells expressing Lv-shNC and Lv-shTMEM44-AS1, as well as KYSE150 cells expressing Vector and Lv-OE (2 × 106) subcutaneously injected into the back of female BALB/cJGpt-Foxn1nu/Gpt nude mice (five mice per group) aged 5 weeks. Measure the tumor volume with a vernier caliper every 4 days. 24 days after injection, euthanize the mice and measure the weight and volume of the tumor. Finally, the tumor was embedded in paraffin and sliced for HE or IHC staining. This experiment was approved by the Biomedical Ethics Committee of the First Affiliated Hospital of First Affiliated Hospital of Zhengzhou University (2022-KY-0971).

### Statistics

All bioinformatics analyses were performed in R version 4.0.2, and all experimental data analysis was carried out in GraghPad Prism 9.0 software. The significance of the differences between the groups was assessed by the Student’s *t* test. Correlations between TMEM44-AS1 and GPX4 gene expression were analyzed by spearman correlation analysis. All data were recorded as Mean ± SD. Survival curves were analyzed by the Kaplan–Meier method and compared by the log-rank test. Statistical significance was determined as *P < 0.05, **P < 0.01, ***P < 0.001, ****P < 0.0001, or no significance (ns).

### Supplementary information

Supplemental Material

Supplemental Material-Original Data

## Data Availability

Data are available from the corresponding author upon reasonable request.
