# Supplementary material for: TMEM44-AS1 promotes esophageal squamous cell carcinoma progression by regulating the IGF2BP2-GPX4 axis in modulating ferroptosis
Source: Cell Death Discov. 2023 Dec 1;9:431. doi: 10.1038/s41420-023-01727-0 (PMC10692126; doi:10.1038/s41420-023-01727-0)
Supplement: Supplementary file 1 — Supplemental Material [file 41420_2023_1727_MOESM1_ESM.pdf]

Supplementary Materials

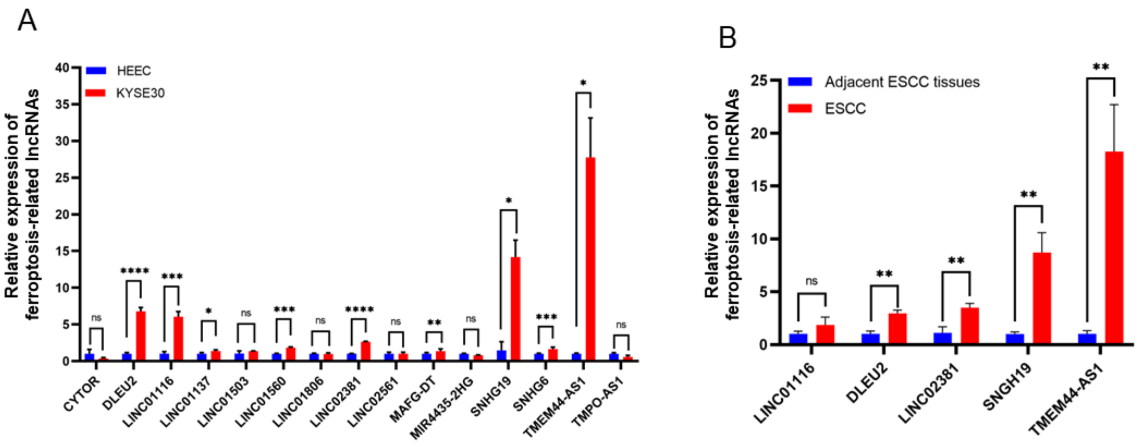

Supplementary Figure S1. Validation of ferroptosis-related lncRNAs in esophageal cancer.

A qRT-PCR analysis of ferroptosis-related lncRNAs expression in ESCC cells. **B** qRT-PCR analysis of ferroptosis-related lncRNAs expression in esophageal cancer tissues. *$p < 0.05$, **$p < 0.01$, ***$p < 0.001$, ****$p < 0.0001$
