# Supplementary material for: TMEM44-AS1 promotes esophageal squamous cell carcinoma progression by regulating the IGF2BP2-GPX4 axis in modulating ferroptosis
Source: Cell Death Discov. 2023 Dec 1;9:431. doi: 10.1038/s41420-023-01727-0 (PMC10692126; doi:10.1038/s41420-023-01727-0)

**Full and uncropped western blot for Figure 3B**

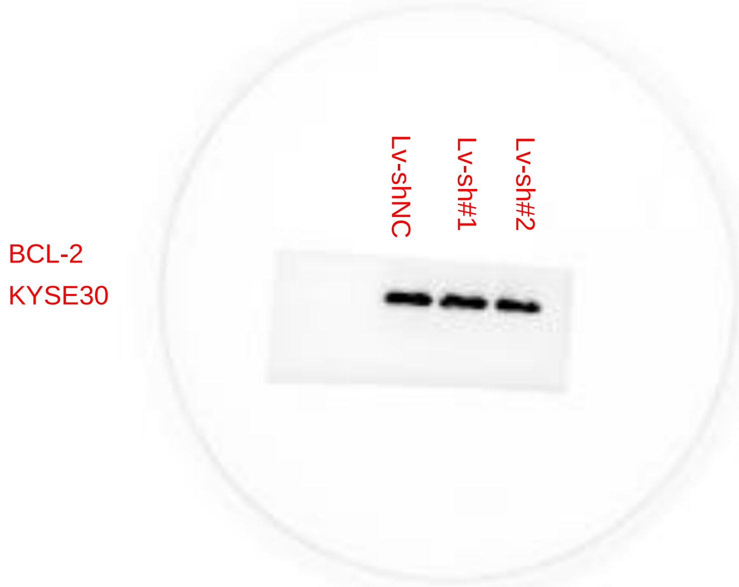

**Full and uncropped western blot for Figure 3B**

Bax
KYSE30

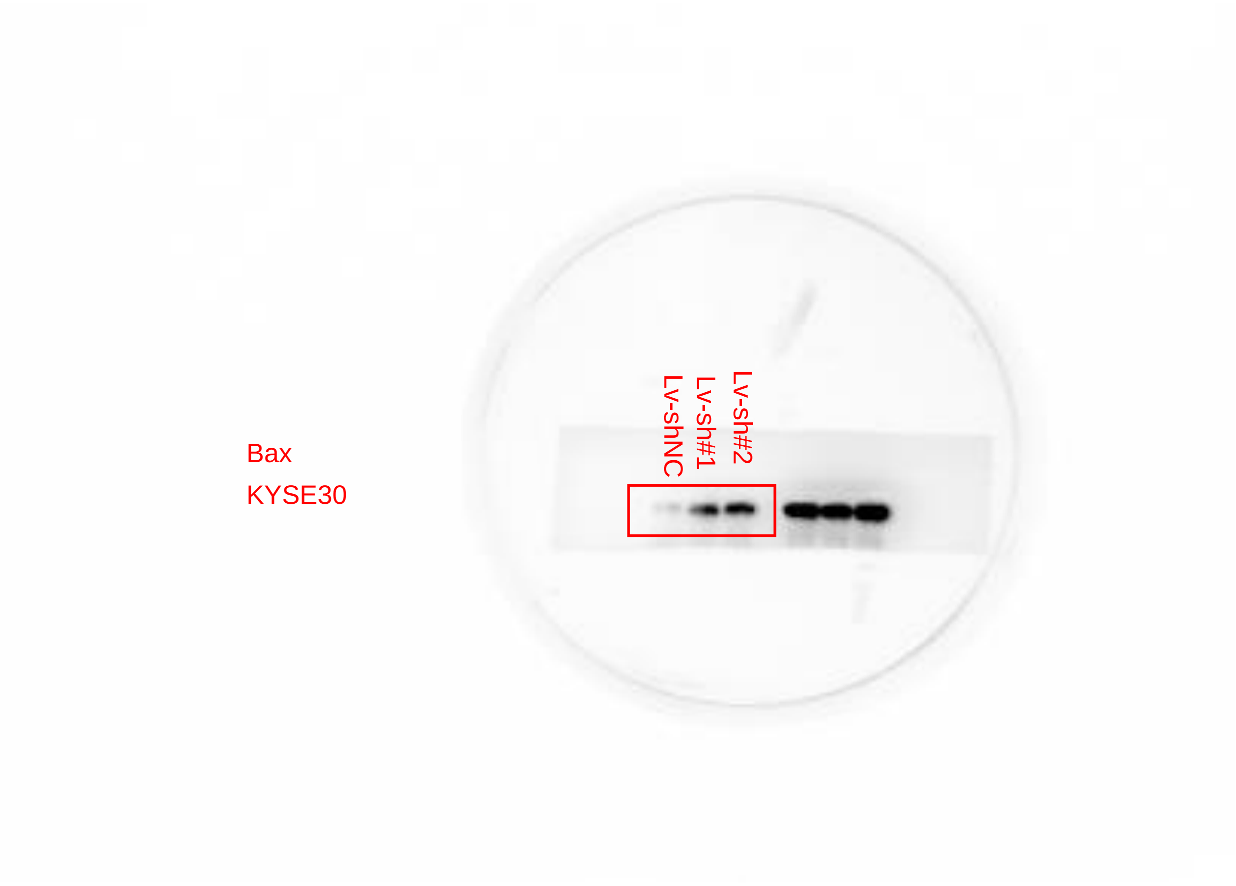


Caspase3
KYSE30

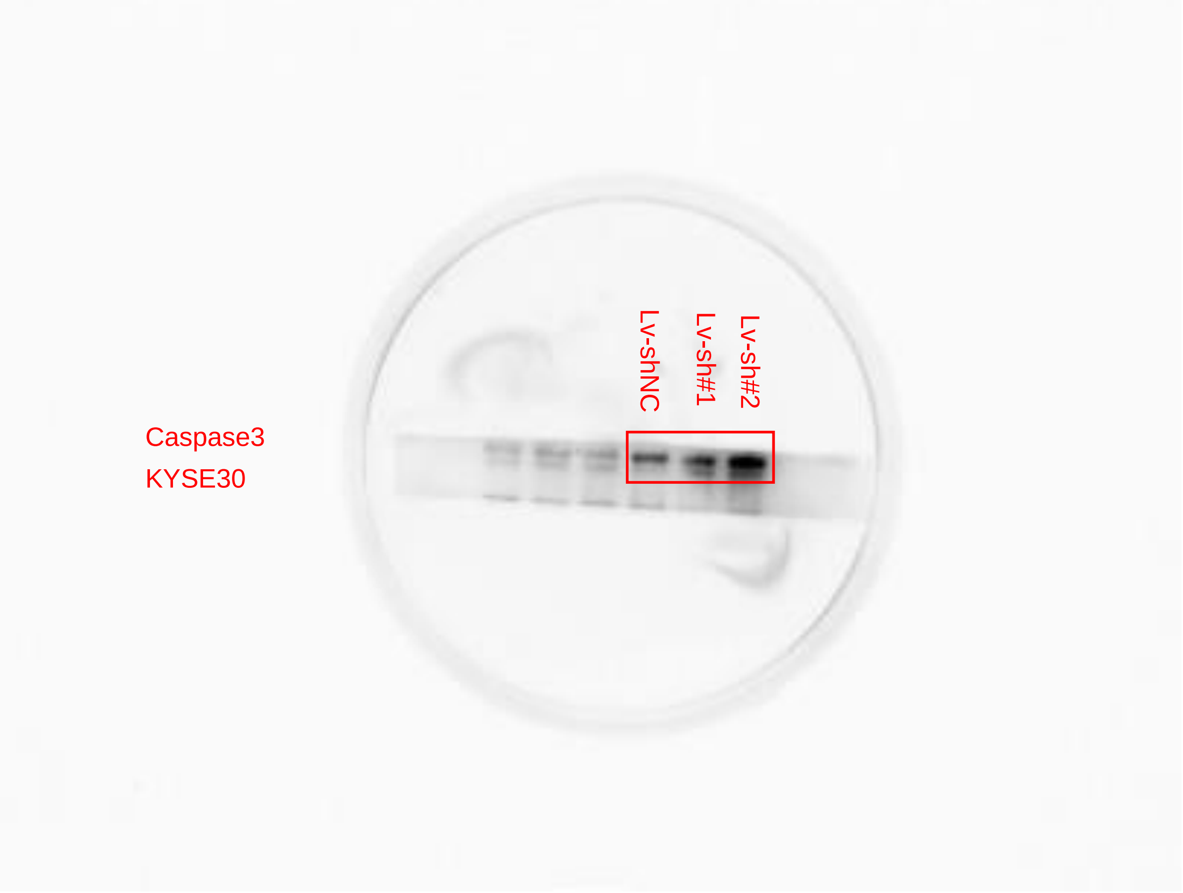

## Full and uncropped western blot for Figure 3B

GAPDH
KYSE30

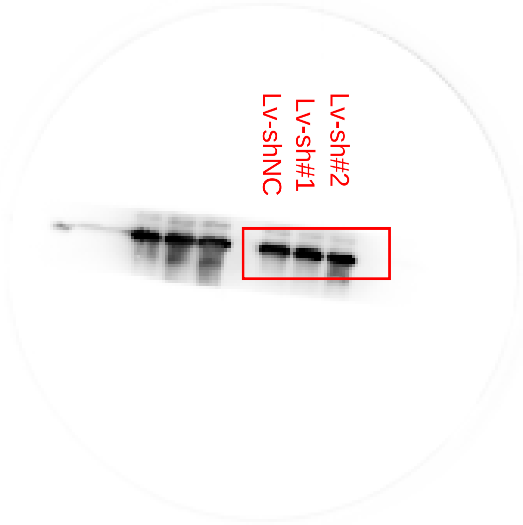


Bcl-2
KYSE150

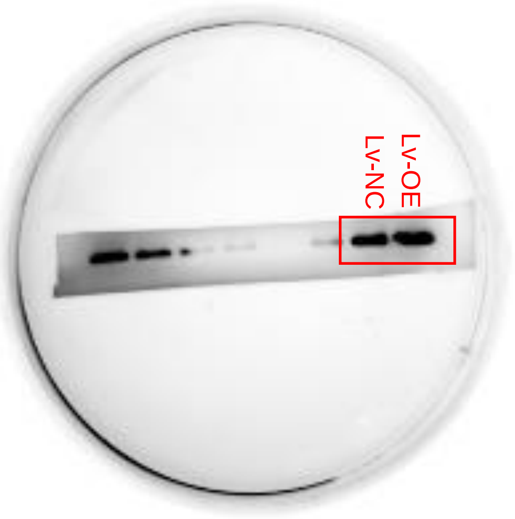

**Full and uncropped western blot for Figure 3B**

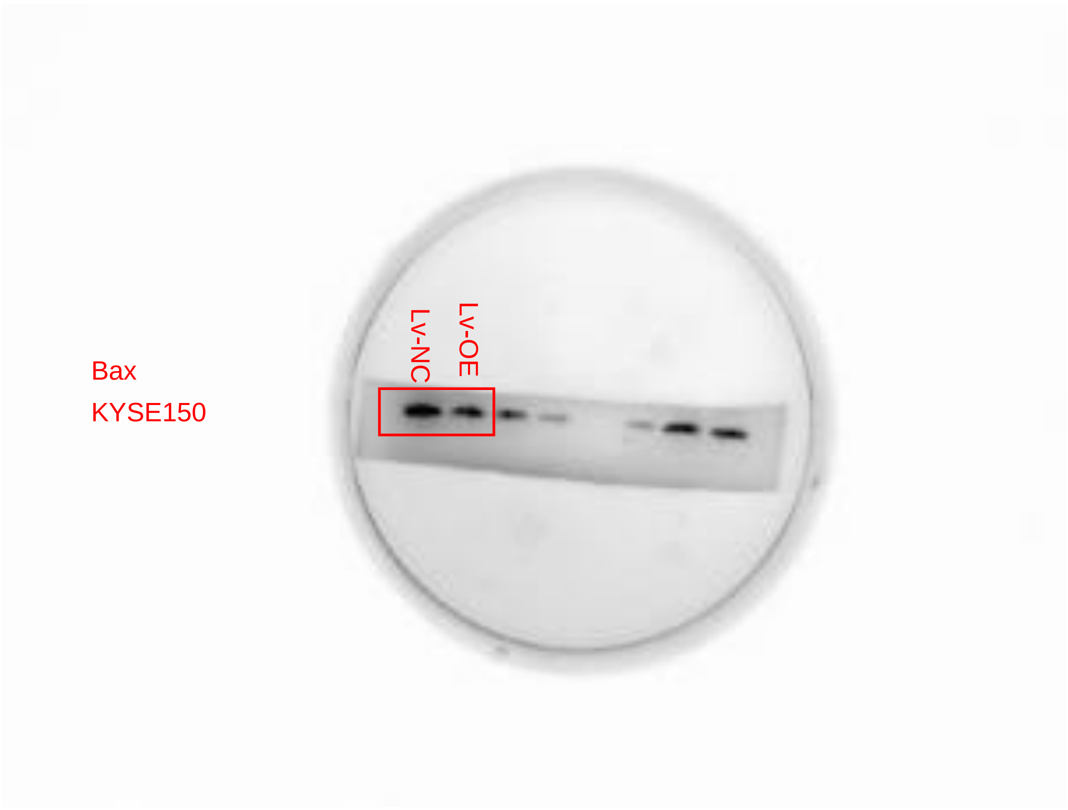


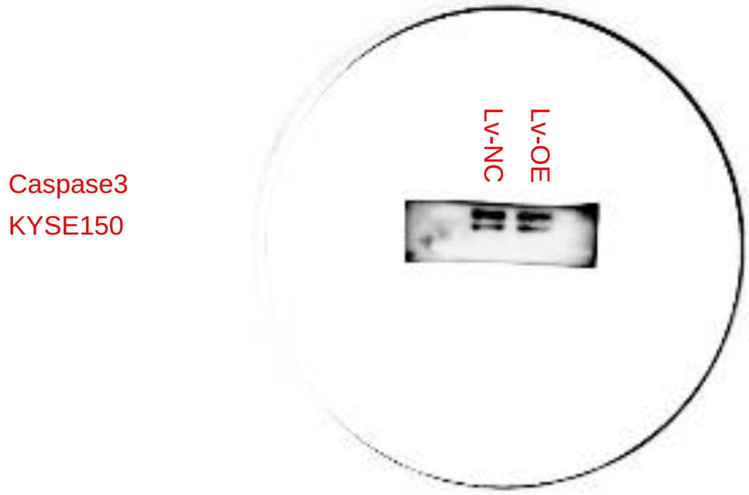

**Full and uncropped western blot for Figure 3B**

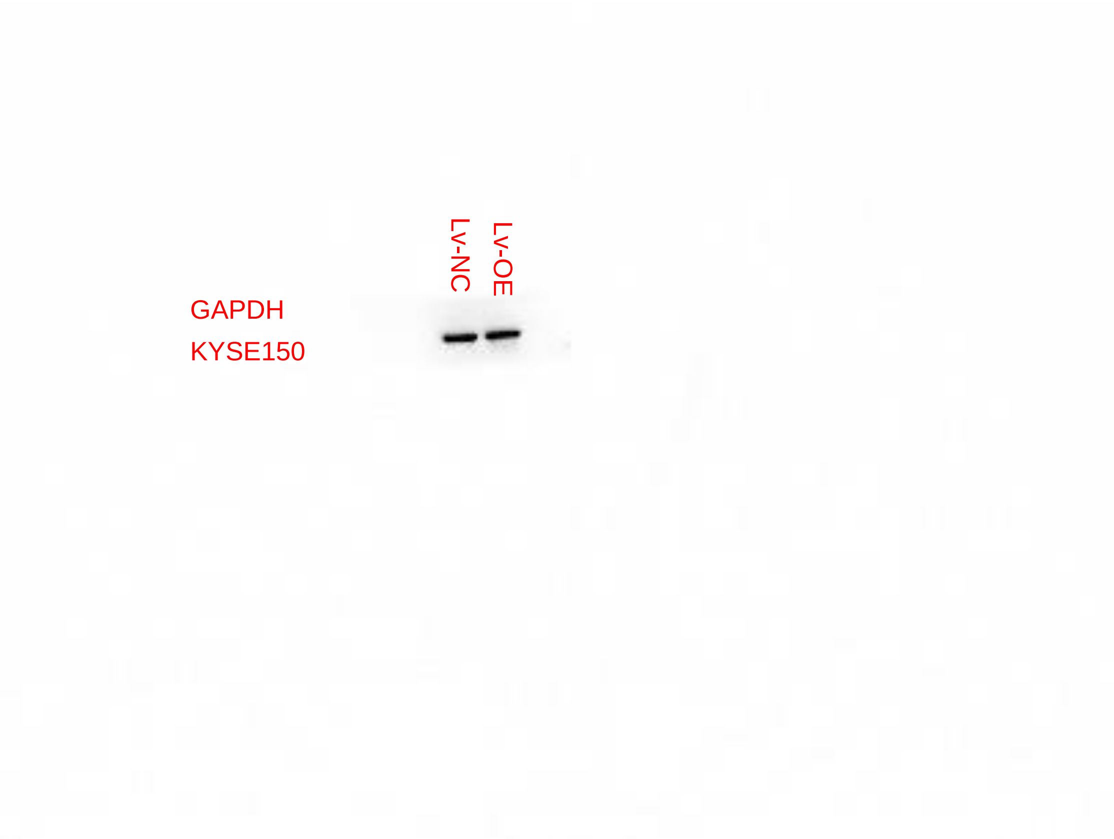


**Full and uncropped western blot for Figure 3C**

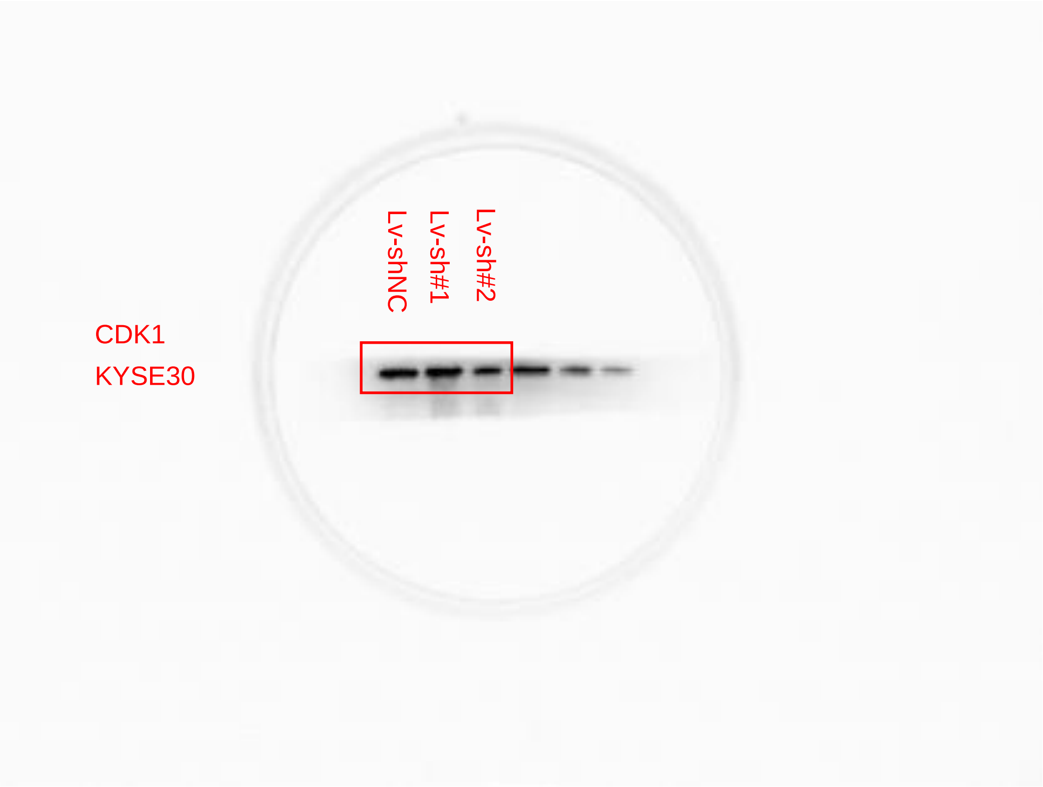

**Full and uncropped western blot for Figure 3C**

CDK6
KYSE30

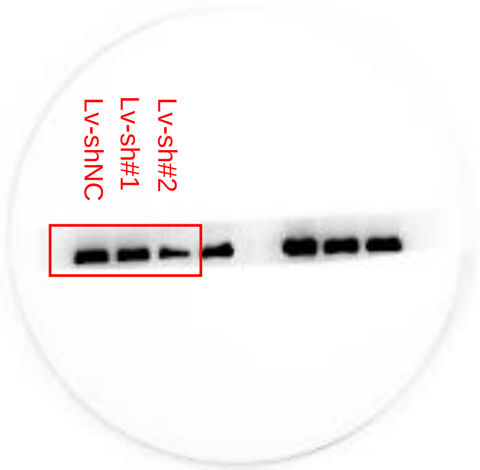


CyclinB1
KYSE30

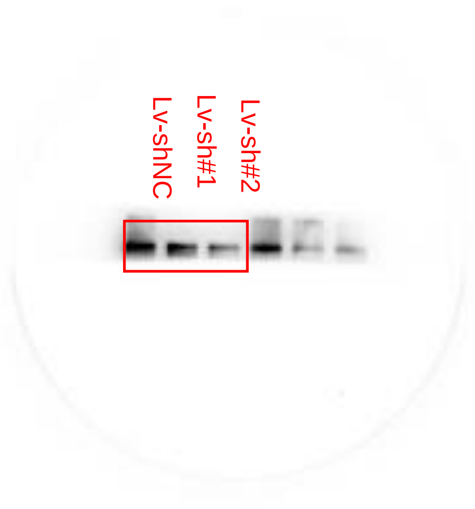

**Full and uncropped western blot for Figure 3C**

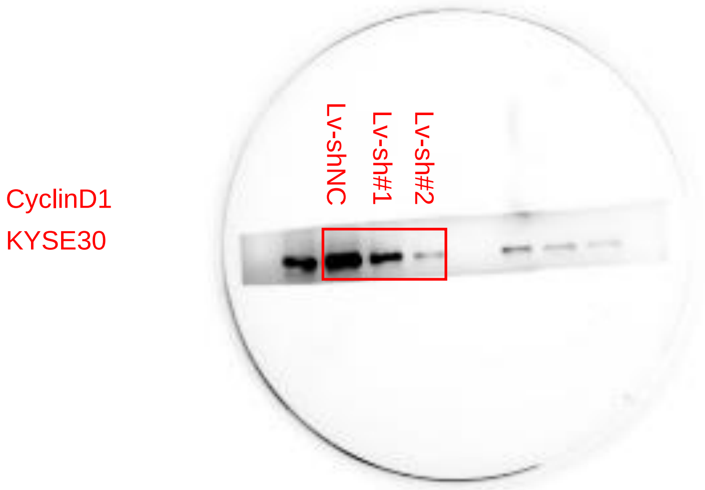


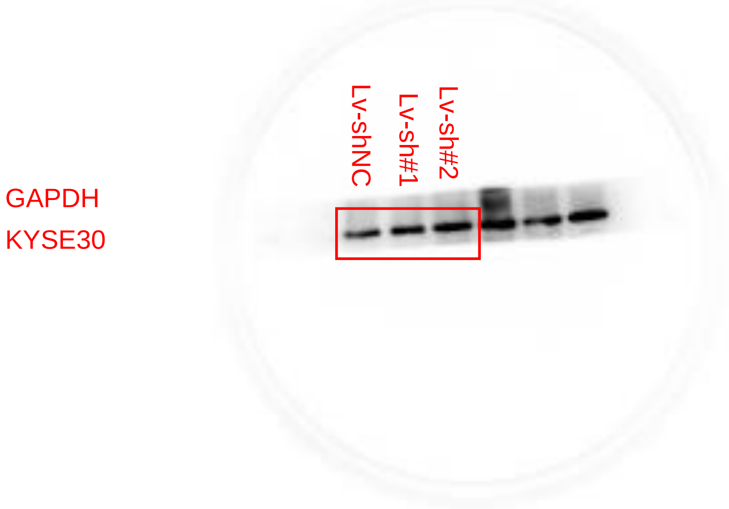

**Full and uncropped western blot for Figure 3C**

CDK1
KYSE150

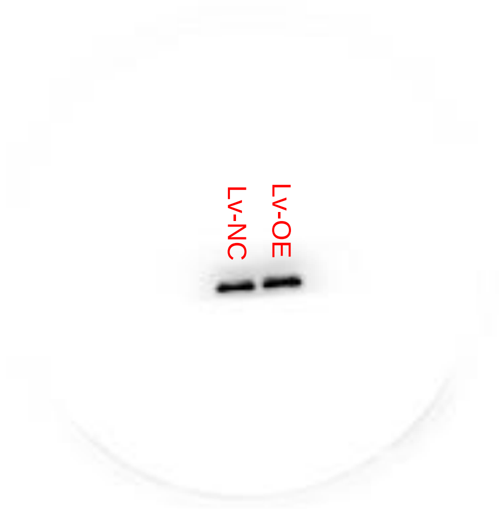


CDK6
KYSE150

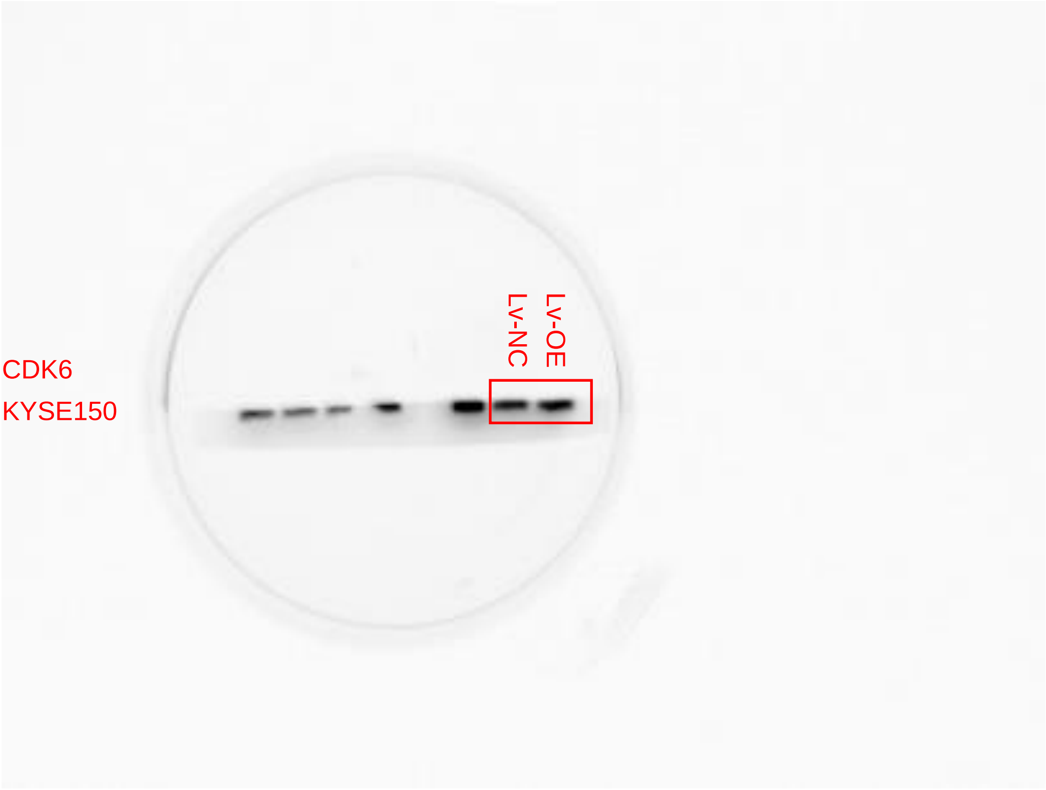

**Full and uncropped western blot for Figure 3C**

CyclinB1
KYSE150

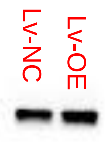


CyclinD1
KYSE150

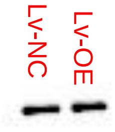

**Full and uncropped western blot for Figure 3C**

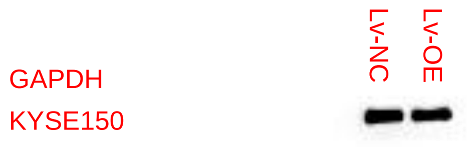


**Full and uncropped western blot for Figure 4A**

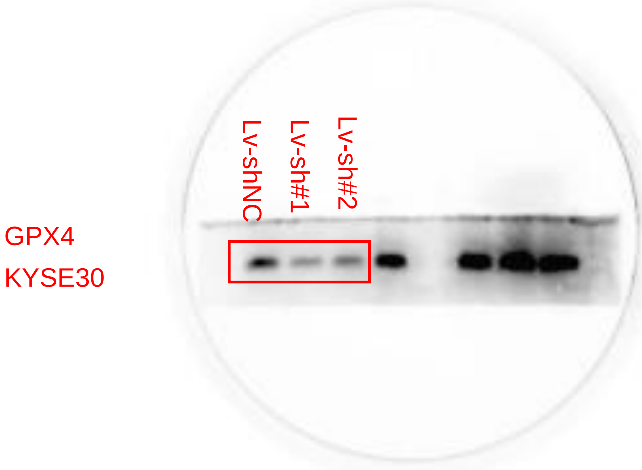

**Full and uncropped western blot for Figure 4A**

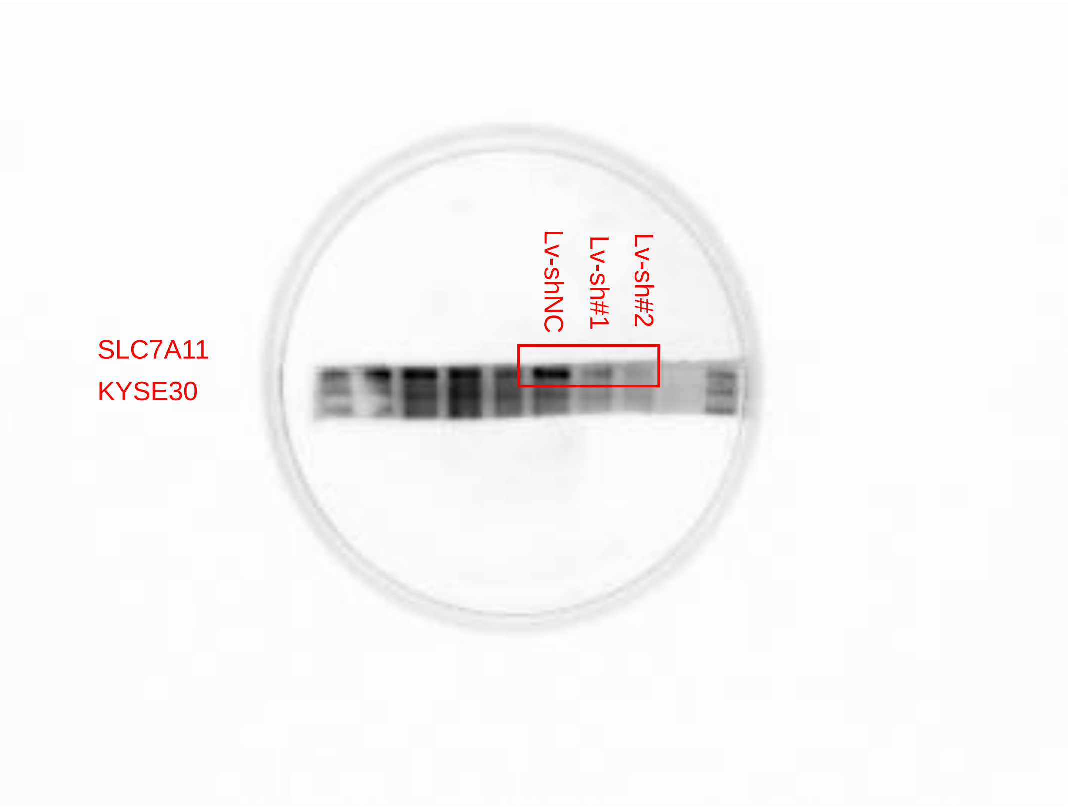


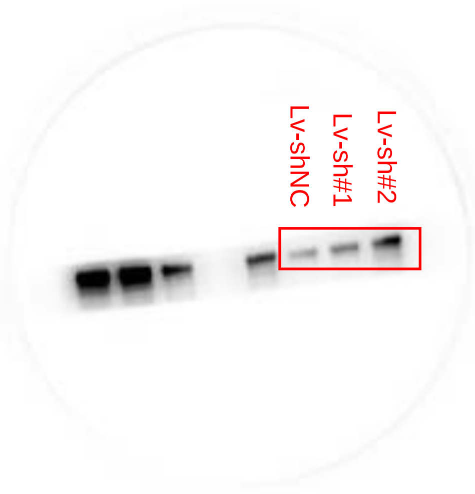

**Full and uncropped western blot for Figure 4A**

P53
KYSE30

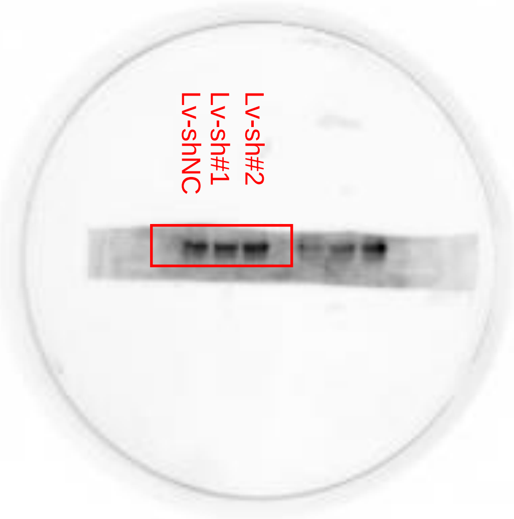


COX2
KYSE30

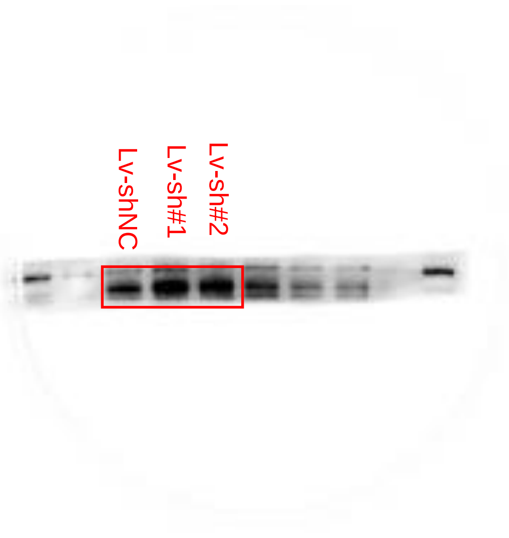

**Full and uncropped western blot for Figure 4A**

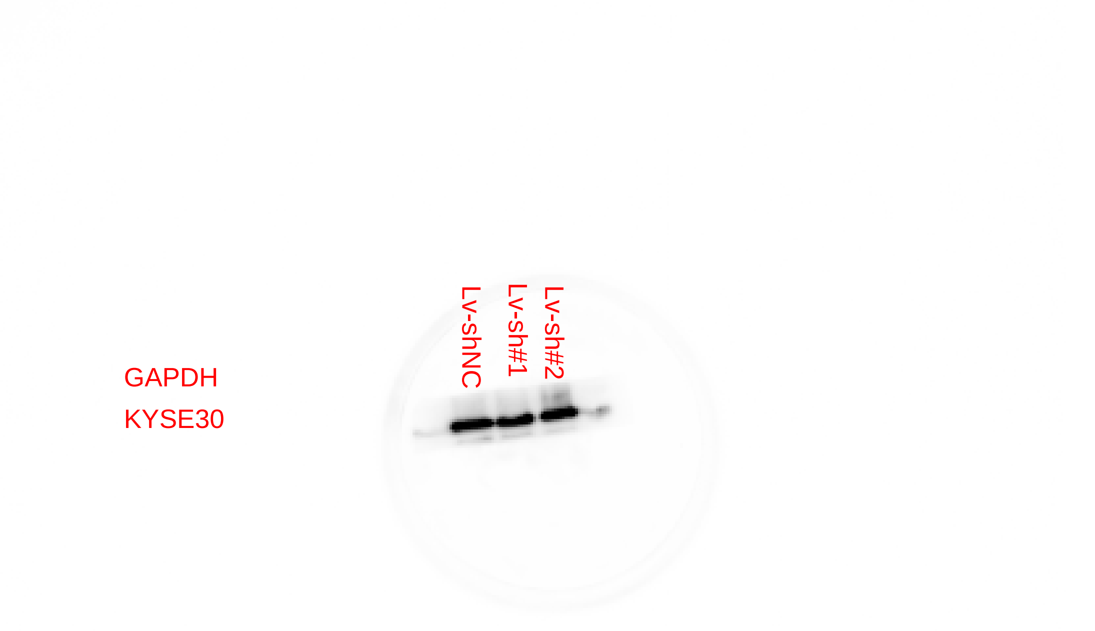


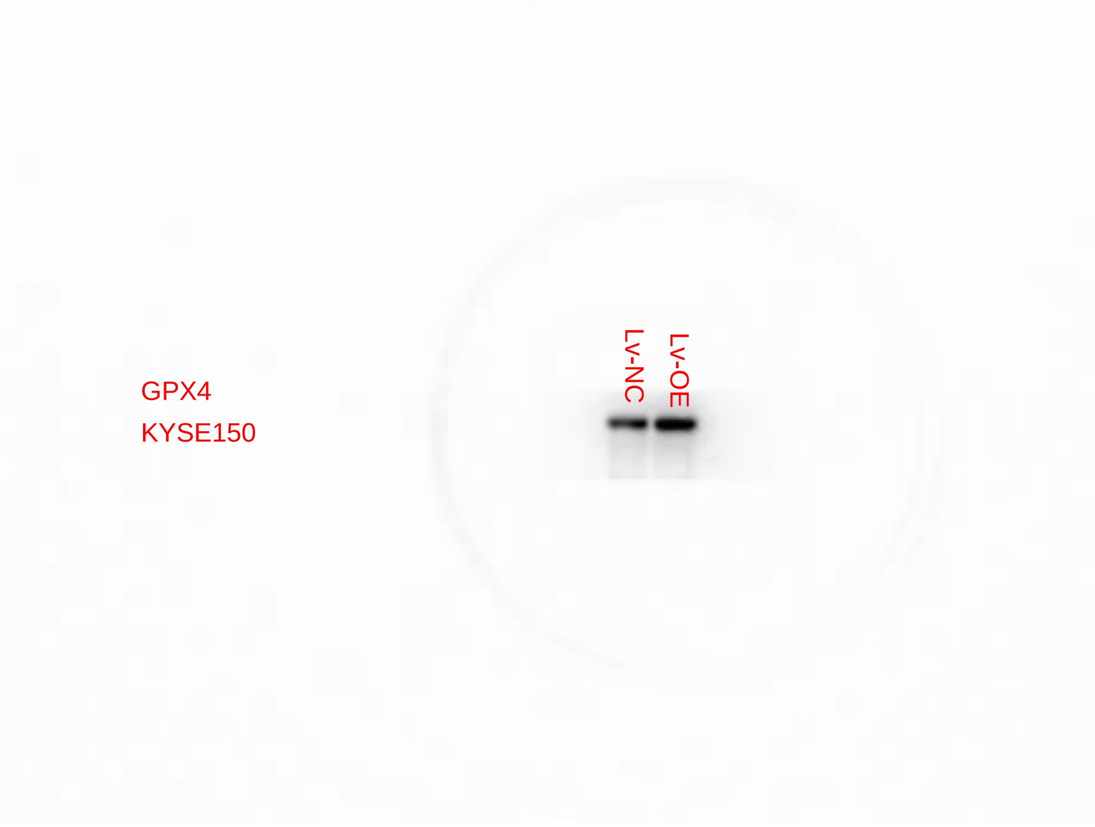

**Full and uncropped western blot for Figure 4A**

SLC7A11
KYSE150

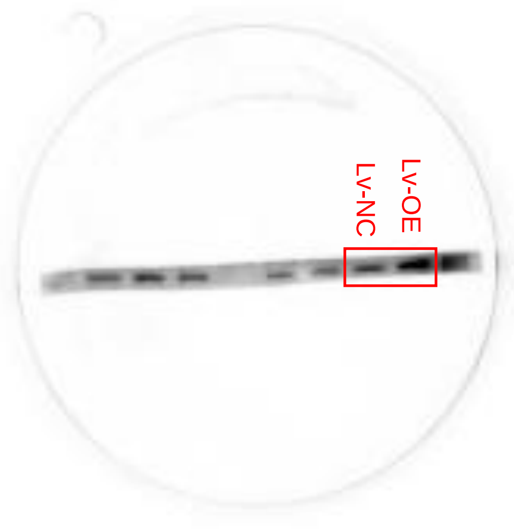


ACSL4
KYSE150

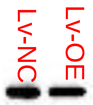

**Full and uncropped western blot for Figure 4A**

P53
KYSE150

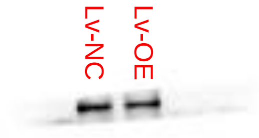


GAPDH
KYSE150

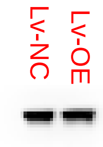

**Full and uncropped western blot for Figure 4G**

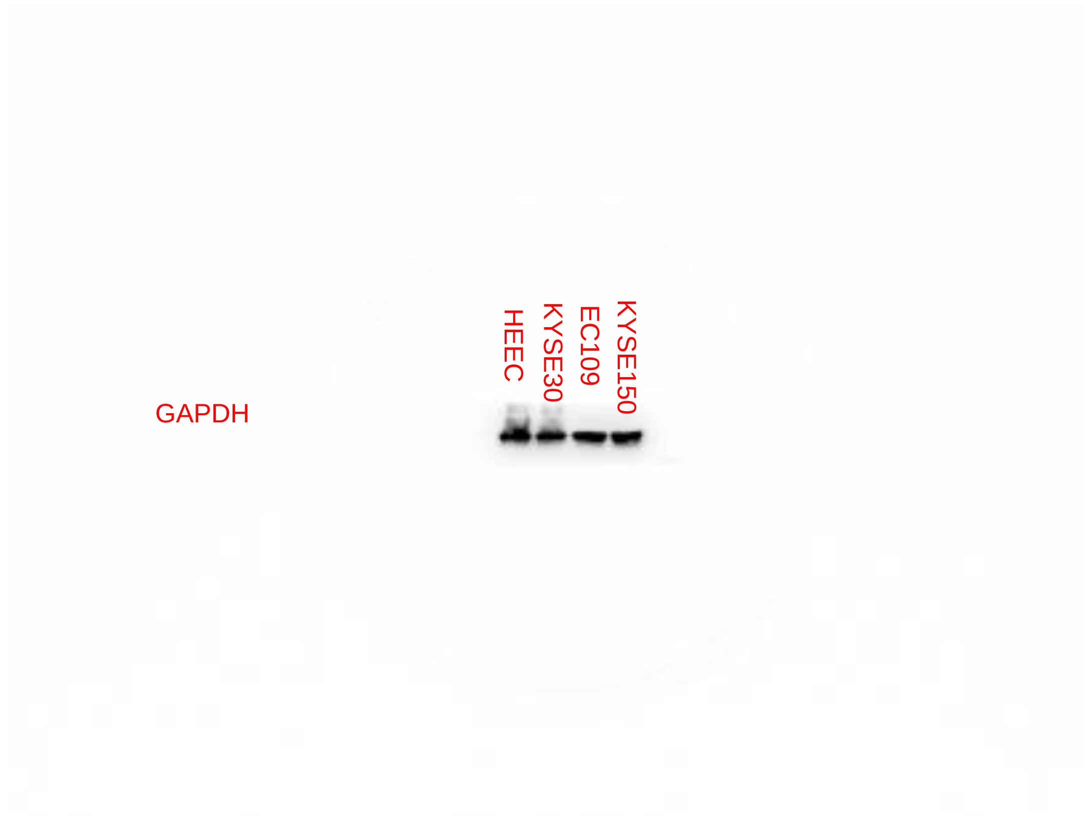


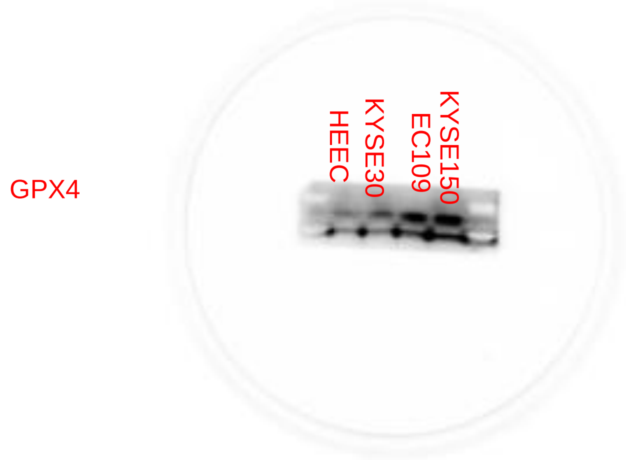

**Full and uncropped western blot for Figure 5E**

Input

GAPDH
KYSE30

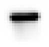

Input Sense Antisense

IGF2BP2
KYSE30

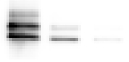

**Full and uncropped western blot for Figure 5E**

Input

GAPDH
KYSE150

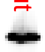

Input Sense Antisense

IGF2BP2
KYSE150

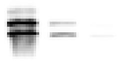

**Full and uncropped western blot for Figure 5F**

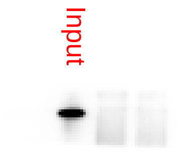


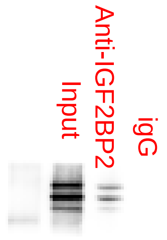

**Full and uncropped western blot for Figure 5F**

GAPDH
KYSE150

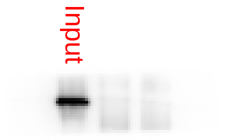


IGF2BP2
KYSE150

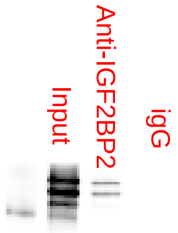

Supplement: Supplementary file 2 — Supplemental Material-Original Data [file 41420_2023_1727_MOESM2_ESM.pdf]
